# Tailored Biocompatible Polyurethane-Poly(ethylene glycol) Hydrogels as a Versatile Nonfouling Biomaterial

**DOI:** 10.1002/adhm.202201378

**Published:** 2022-08-29

**Authors:** Alessondra T. Speidel, Phillip R. A. Chivers, Christopher S. Wood, Derrick A. Roberts, Inês P. Correia, April S. Caravaca, Yu Kiu Victor Chan, Catherine S. Hansel, Heimgärtner Johannes, Eliane Müller, Jill Ziesmer, Georgios A. Sotiriou, Peder S. Olofsson, Molly M. Stevens

**Affiliations:** Department of Medical Biochemistry and Biophysics Karolinska Institutet Stockholm 171 77, Sweden; Key Centre for Polymers and Colloids School of Chemistry The University of Sydney Sydney, NSW 2006, Australia; Department of Medical Biochemistry and Biophysics Karolinska Institutet Stockholm 171 77, Sweden; Laboratory of Immunobiology Stockholm Center for Bioelectronic Medicine Department of Medicine, Solna Karolinska Institutet Stockholm 171 77, Sweden; Department of Medical Biochemistry and Biophysics Karolinska Institutet Stockholm 171 77, Sweden; Science for Life Laboratory Department of Medical Biochemistry and Biophysics Karolinska Institutet Stockholm 171 77, Sweden; Department of Medical Biochemistry and Biophysics Karolinska Institutet Stockholm 171 77, Sweden; Department of Microbiology Tumor and Cell Biology Karolinska Institutet Stockholm 171 77, Sweden; Laboratory of Immunobiology Stockholm Center for Bioelectronic Medicine Department of Medicine, Solna Karolinska Institutet Stockholm 171 77, Sweden; Center for Biomedical Science and Bioelectronic Medicine The Feinstein Institute for Medical Research Manhasset, NY 11030, USA; Department of Medical Biochemistry and Biophysics Karolinska Institutet Stockholm 171 77, Sweden; Materials Department of Bioengineering and Institute for Biomedical Engineering Imperial College London London SW7 2AZ, UK

**Keywords:** biomaterials, hydrogels, nonfouling, polyethylene glycol (PEG), polyurethane

## Abstract

Polyurethane-based hydrogels are relatively inexpensive and mechanically robust biomaterials with ideal properties for various applications, including drug delivery, prosthetics, implant coatings, soft robotics, and tissue engineering. In this report, a simple method is presented for synthesizing and casting biocompatible polyurethane-poly(ethylene glycol) (PU-PEG) hydrogels with tunable mechanical properties, nonfouling characteristics, and sustained tolerability as an implantable material or coating. The hydrogels are synthesized via a simple one-pot method using commercially available precursors and low toxicity solvents and reagents, yielding a consistent and biocompatible gel platform primed for long-term biomaterial applications. The mechanical and physical properties of the gels are easily controlled by varying the curing concentration, producing networks with complex shear moduli of 0.82–190 kPa, similar to a range of human soft tissues. When evaluated against a mechanically matched poly(dimethylsiloxane) (PDMS) formulation, the PU-PEG hydrogels demonstrated favorable nonfouling characteristics, including comparable adsorption of plasma proteins (albumin and fibrinogen) and significantly reduced cellular adhesion. Moreover, preliminary murine implant studies reveal a mild foreign body response after 41 days. Due to the tunable mechanical properties, excellent biocompatibility, and sustained in vivo tolerability of these hydrogels, it is proposed that this method offers a simplified platform for fabricating soft PU-based biomaterials for a variety of applications.

## 1 Introduction

Hydrogels are popular materials in biomedical engineering with myriad applications in drug delivery,^[1]^ prosthetics and implants,^[2]^ tissue engineering,^[3^–^5]^ and soft robotics.^[6]^ The similarity in water content between hydrogels and soft tissues, as well as the tunability of their mechanical and physical properties, make these materials especially attractive for constructing implantable devices or tissue engineering scaffold materials that interface well with surrounding tissues. Among the plethora of synthetic and natural polymers that have been used to prepare hydrogel biomaterials, polyurethanes are receiving growing attention due to their excellent mechanical properties, good biocompatibility, and emerging methods to tune their degradability.^[7–9]^

Polyurethanes (PUs) are characterized by urethane, or carbamate, linkages along their backbones, which are chemically related to the amide bonds in proteins. The capacity of polyurethanes to form hydrogen bonds is a key toughening mechanism that underpins their high mechanical strength.^[10]^ By combining hydrophobic polyurethanes with water-soluble polymers, it is possible to generate swellable copolymer networks that are robust and durable while still exhibiting soft hydrophilic properties ideal for interfacing with, and even possibly eventually replacing, biological tissues.^[2–5]^ Despite their growing utility, there is a general perception that commercial polyurethane precursors (e.g., isocyanates) are diffcult to handle due to their high reactivity and moisture sensitivity.^[11–13]^ Moreover, standard PU synthesis procedures typically employ toxic tin(II) catalysts and can employ harmful organic solvents (e.g., *N,N*-dimethylformamide (DMF)) in synthetic methods commonly used in laboratory settings. Although suffcient removal of these reactants is easily possible with traditional commercial polyurethane coatings, in the synthesis of copolymerized bulk materials, retention of these components can reduce biocompatibility of the resulting materials if not removed completely.^[14,15]^ While alternative isocyanate-free syntheses of polyurethanes are being developed,^[16^–^19]^ the use of noncommercial reagents can limit the ease of fabrication. Furthermore, while less toxic commercial reagents can, in principle, be used to make polyurethane biomaterials, in practice, toxic reagents and solvents remain remarkably prevalent in contemporary biomaterials literature.^[4,20–22]^

In this study, we report a simple one-pot method for synthesizing and casting polyurethane-poly(ethylene glycol) (PU-PEG) hydrogels. Our approach focuses on the use of dimethyl sulfoxide (DMSO) and 1,4-diazabicyclo[2.2.2]octane (DABCO) as less toxic and greener alternatives to the solvents and catalysts more commonly used for polyurethane synthesis (e.g., DMF and dibutyltin dilaurate, DBTDL).^[23–25]^ We also describe a simple casting apparatus that can be fashioned from inexpensive laboratory consumables, enabling the preparation of gel pucks and sheets to suit different applications. Importantly, our fabrication methods are optimized for operators with access to common equipment and procedures available in most laboratories.

The simple and robust methodology reported herein can be used to prepare materials with a wide range of structural and functional properties, simply by varying the concentration of the reaction components. Such versatility could enable the facile fabrication of materials mimicking key properties of a wide range of biological tissues, which, in turn, may improve their integration in vivo either as parts of tissue engineering materials or as part of a medical device. Herein, we demonstrate that mechanical properties spanning several orders of magnitude can be accessed using our synthetic procedure, and we explore how these material properties determine their performance in vitro and in vivo. Specifically, we have evaluated the cytotoxicity, nonfouling characteristics and foreign body response (FBR) of our materials to demonstrate their potential as biomaterials in a wide range of applications. By demonstrating simple casting conditions, facile modular mechanical and physical properties, improved biocompatibility, and implant tolerability, we hope to extend a simplified platform for tailored PU-based hydrogels for various biomaterials applications.

## 2 Results and Discussion

### 2.1 PU-PEG Hydrogel Preparation

**Figure 1:**
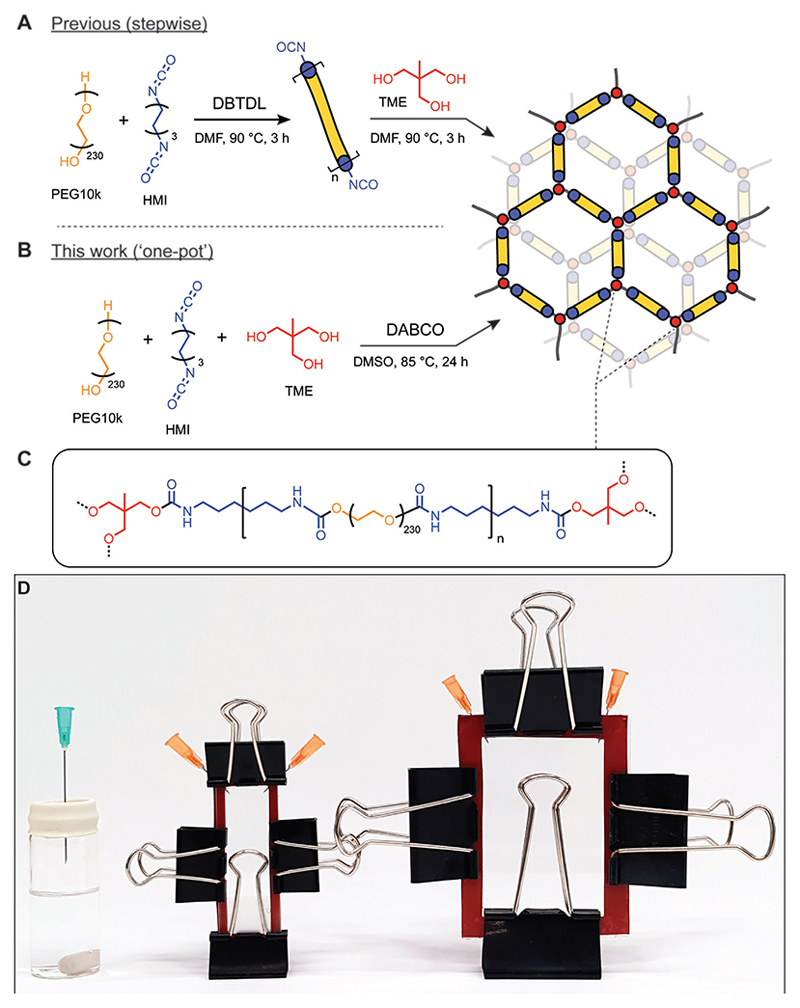
One-pot synthesis and reusable casting set-up for PU-PEG hydrogels. A) Previously reported stepwise synthesis of PU-PEG hydrogel network.^[27]^ B) Optimized one-pot synthesis of PU-PEG hydrogel network combining synthetic steps, substituting greener reagents, including DMSO in place of DMF as reaction solvent, and organic basic catalyst DABCO for the commonly used cytotoxic tin-containing DBTDL catalyst. C) A stylized depiction of the chemical structure of the final PU-PEG hydrogel network. D) Images of the various affordable hydrogel synthesis casting set-ups presented in this work to generate final hydrogel forms of different shapes and volumes. Reaction mixture injected through one needle into each set up. One needle in the flat gel casting set-ups vents displaced gas as the reaction solution is injected. Silicone gasket spacers (red) serve to generate gels of desired thicknesses and can be cut out from sheets purchased cheaply at a range of desired thicknesses. Binder clips hold the configurations in air-tight conformations. (Left) Vial configuration, (middle) microscope slide sheet configuration, (right) western blot glass sheet configuration

PU-PEG hydrogels are commonly fabricated through a two-step synthesis that uses the toxic catalyst, DBTDL, and solvent, DMF (Figure 1A).[2,26–28] In this work, we have shown that this synthesis may be condensed into one stage and demonstrated successful substitution of less toxic reagents, employing the organic base DABCO as a catalyst, and DMSO as a solvent (Figure 1B).^[25]^ The simplified one-pot synthesis allows for the combination of reagents in a single step, reducing the opportunities to introduce compositional variability (Figure 1).^[27]^ In a typical procedure, dried PEG (MW 10 kDa, 0.2 mmol), tris(hydroxymethyl)ethane (TME, 0.28 mmol) crosslinker and either DABCO or DBTDL catalyst (0.014 mmol) were dissolved with heating in anhydrous solvent (DMSO, DMF, or ACN) under an inert atmosphere. Hexamethylene diisocyanate (HMI, 1.4 mmol) was added to the mixture, which was then cured at 85 °C for 24 h. This method enabled the consistent formation of PU-PEG gels, which were washed sequentially with tetrahydrofuran (THF) and water to remove unreacted components and yield robust hydrogels.

The substitution of less toxic reagents engenders a more sustainable synthetic approach and reduces the likelihood that resulting materials retain any cytotoxic precursors, which would be of particular importance in the creation of any biomaterials that are intended for long-term implantation. First, the traditional use of a tin-containing DBTDL catalyst in PU-PEG hydrogel synthesis^[27,28]^ (Figure 1A), introduces a potential source of cytotoxicity in instances where the catalyst cannot be completely washed out of the cured gels. In this work, a water-soluble organic base catalyst, DABCO, has been substituted for DBTDL, which is insoluble in water (Figure 1B). Similarly, synthesis of the gels was found to be compatible with solvents less toxic than DMF, including acetonitrile and DMSO.^[25,27]^ By contrast, attempts to prepare the gels in THF were unsuccessful (Table S1, Supporting Information).

The concentration of reactants also had a significant influence on the curing rate. Qualitatively, it was observed that gels prepared at 46% (w/v) formed after only 2 h, whereas 23% (w/v) and 12% (w/v) cured after 5 and 24 h, respectively. Intuitively, time to gelation is slower at lower reactant concentrations; the reaction proceeds more slowly and a greater proportion of the polymer chains must be crosslinked before the solution is gelled.^[29]^ To further probe the reaction processes underlying gel curing, we used IR spectroscopy to follow HMI consumption via reduction in isocyanate peak intensity (≈2270 cm^–1^, Figure S1, Supporting Information) and concomitant growth of resonances corresponding to the formation of urethane carbonyl groups (Figure S2, Supporting Information). As expected, the rate of HMI consumption broadly correlates with reactant concentration and at all concentrations, the isocyanate band was no longer visible after ≈3 h. This decrease to baseline noise correlates to ≈90%, 80%, and 63% HMI conversion for reactions at 46% (w/v), 23% (w/v) and 12% (w/v). Evidence of urethane formation occurred on a slightly slower timescale. C=O stretches at 1715 cm^–1^ reached a maximum intensity after 3–5 hinall samples, suggesting that the reaction between HMI and PEG was complete. A second, smaller peak in the carbonyl region (≈1681 cm^–1^) grows on a timescale more consistent with gelation for the respective samples, which we attribute to urethane bonds formed by crosslinking reactions between HMI and hydroxyl groups of TME. Alternatively, it may arise from oligo-urethane moieties formed by competing reactions of urethane nitrogen atoms with isocyanate.^[30]^ IR spectra of wet gel samples after sequential THF and water washing steps showed a large, broad peak at 1633 cm^–1^. This may represent hydrogen-bonded polyurea carbonyl stretches formed by hydrolysis of residual HMI isocyanate groups,^[31]^ although the concentration independence of this peak’s intensity warrants further investigation before a concrete assignment can be made.

To assist in the preparation of gels with more appropriate dimensions for materials characterization and animal studies, we aimed to establish curing set-ups which would enable the fabrication of gel sheets. Further, we aimed to make these casting setups reusable and simple to assemble from materials and equipment common in standard biomaterials laboratories. The presented simplified gel casting set-ups implement cheap and commercially available materials along with commonly available and reusable glass components. Parallel glass plate set-ups were assembled from glass microscope slides (Figure 1D, middle) or larger western blot glass plates (Figure 1D, right) separated with a silicone gasket and held tightly together with binder clips (Figure 1D), subsequently referred to as the “sheet configuration.” The thickness of the final gels can be easily adjusted through the silicone gasket thickness; we successfully cast gels varying from 0.5 to 1.5 mm in thickness. To prepare larger and thicker gels, a glass vial sealed with a rubber stopper was used as a curing mold. This is referred to here as the “vial configuration” (Figure 1D, left). It was shown that the one-pot synthesis could be performed reproducibly for both the vial and sheet configurations. Challenges associated with keeping reagents and casting set-ups free of water could be anticipated, but the synthesis of PU-PEG hydrogels was possible without the need for more complex air exclusion techniques.

### 2.2 Tunability of Hydrogel Mechanical and Physical Properties

**Figure 2:**
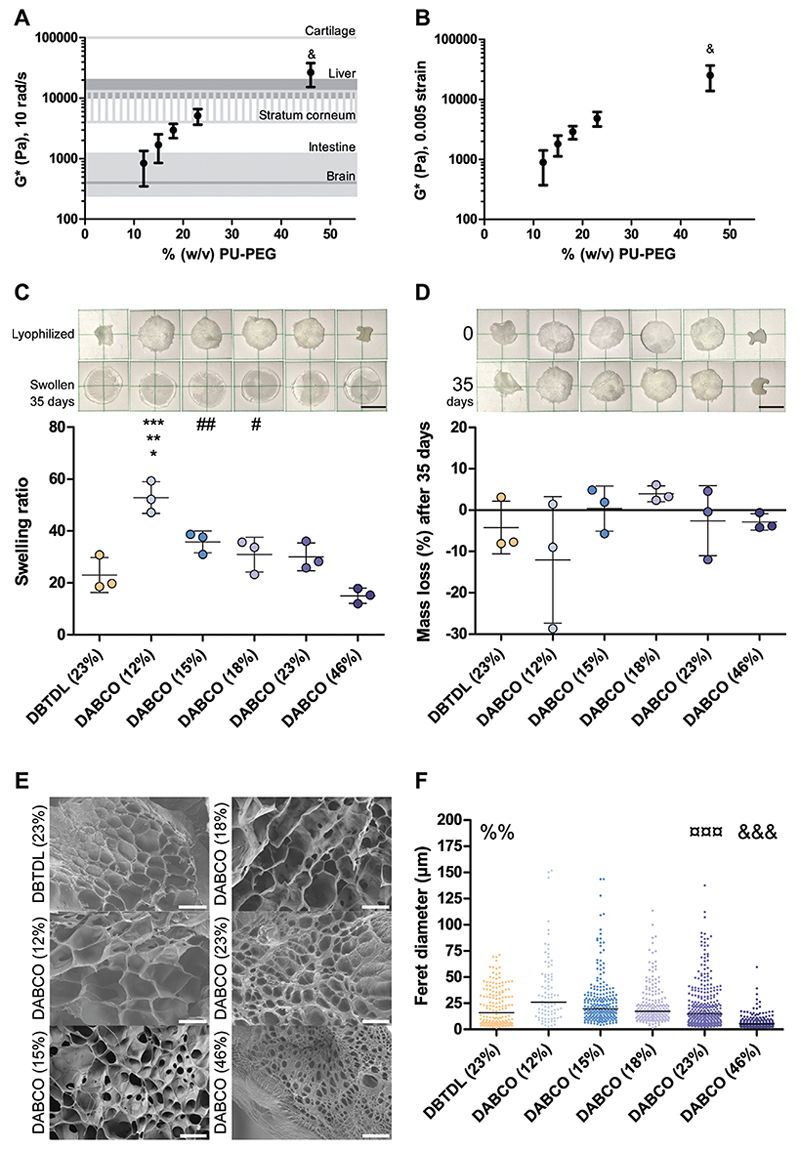
Tunable PU-PEG hydrogel mechanical and physical properties Complex shear modulus of 12% (w/v), 15% (w/v), 18% (w/v), 23% (w/v), and 46% (w/v) PU-PEG DABCO-catalyzed hydrogels at A) 10 rad/s and B) 0.005 strain compared with human brain,^[32]^ intestine,^[33]^ stratum corneum,^[34]^ liver,^[35]^ and cartilage.^[36]^ C) Swelling ratio and D) mass loss of 23% (w/v) DBTDL-catalyzed and 12% (w/v), 15% (w/v), 18% (w/v), 23% (w/v), and 46% (w/v) DABCO-catalyzed PU-PEG hydrogels after 35 days in 37 °C water. Representative images of samples from each formulation are shown above C) and D). Scale bars are 5 mm. All experiments conducted on gels from 3 to 5 independent syntheses (*n* = 3–5), each with 3–5 replicate gels assessed. Error bars illustrate standard deviation across syntheses. Samples from rheology, swelling ratio, and degradation analysis compared by one-way ANOVA, Tukey post-test. &p<0.05 compared with all other formulations, *p<0.05 with DABCO (15%), ^#^*p*<0.05 with DABCO (46%), **p<0.01 with DABCO (23%, 18%), ^##^*p*<0.01 with DABCO (46%), ***p<0.001 with DABCO (46%, 23%). E) Representative SEM images and F) Feret diameter quantification of freeze fracture lyophilized 23% (w/v) DBTDL-catalyzed and 12% (w/v), 15% (w/v), 18% (w/v), 23% (w/v), and 46% (w/v) DABCO-catalyzed PU-PEG hydrogels. Scale bars are 50 μm. Median pore size for each formulation indicated with a line (n = 3–6, at least 86 total pores analyzed per formulation). Samples from SEM analysis compared by Kruskal-Wallis Test, Dunn’sMultiple Comparison post-test.%%*p*<0.01 with DABCO (46%, 15%, 12%), &&&*p*<0.0001 compared with all other formulations, ¤¤¤*p*<0.0001 with DABCO (46%, 15%, 12%)

The stoichiometric ratios of PEG, HMI, TME, and DBTDL or DABCO catalyst were consistent with those reported in previous literature,^[27]^ but the relative volume of solvent was varied in order to generate hydrogels at a wide range of concentrations (Table S2, Supporting Information). Modulation of the ratio (% w/v) of PEG, HMI, and TME to solvent in the gelation process facilitated control over the material properties of PU-PEG hydrogels (Figure 2).

### 2.2.1 Mechanical Characterization

PU-PEG gels cast at concentrations of 9.2–223% (w/v) exhibited a complex shear modulus (G*) ranging from 0.82 to 190 kPa (Figure S3, Supporting Information), spanning the mechanical properties exhibited by soft human brain tissue to stiff human cartilage (Figures 2A, B). Human brain tissue has been reported to display G* values around 300–400 Pa,^[32]^ similar to mechanical properties observed by 9.2% (w/v) PU-PEG hydrogels (Figure S3, Supporting Information). The mechanical properties of the 12% (w/v) PU-PEG gels resemble those of human colon.^[33]^ The outer layer of human skin, called the stratum corneum, has been reported to display a G* of 4–12 kPa,^[34]^ resembling the mechanical properties exhibited by the 23% (w/v) PU-PEG gels. The 46% (w/v) PU-PEG gels exhibit mechanical properties on the upper end of those reported for human liver tissue.^[35]^ Human articular cartilage has been reported to display a complex shear modulus (G*) between 0.1 and 2.5 MPa,^[36]^ in line with 223% (w/v) PU-PEG, the most concentrated formulation fabricated (Figure S3, Supporting Information). Mechanical matching of implant materials is implicated in a reduced foreign body response.^[37]^ The demonstrated ability to access a broad range of hydrogel mechanical properties simply by varying the concentration of PU-PEG may therefore have significant implications for the utility of these materials as implantable biomaterials or coatings.

Representative frequency and strain sweeps for every synthesis of each PU-PEG formulation can be found in Figures S4 and S5 (Supporting Information).

### 2.2.2 Swelling and Degradation Behavior

The swelling behavior of the various PU-PEG hydrogel formulations also varied according to the concentration of the gels, with more concentrated hydrogel formulations displaying a diminished swelling capacity (Figure 2C). Mass swelling ratios (q) ranged from 15.06 ± 2.91 to 52.87 ± 6.09 for 46% (w/v) and 12% (w/v) DABCO-catalyzed PU-PEG hydrogels, respectively (Figure 2C). None of the formulations showed any significant degradation over the course of 35 days (Figure 2D).

The full range of swelling and degradation behavior for each formulation over 35 days are displayed in Figures S6 and S7 (Supporting Information), respectively.

### 2.2.3 Porosity

The pore sizes of the PU-PEG hydrogels varied according to hydrogel concentration with the smallest pores in the most concentrated formulations (Figure 2E, F). The median Feret diameters and interquartile ranges of the PU-PEG hydrogels’ pores range from 5.17 µm (IQR: 4.86 µm) to 26.10 µm (IQR: 35.92 µm) for 46% (w/v) to 12% (w/v) DABCO-catalyzed PU-PEG hydrogels, respectively (Figure 2F), in line with previous reports of similar materials in the literature.^[26,38]^ The interquartile ranges illustrate the range of pore sizes that span 25–75% of the distribution (Equation 3). Further images of the gels are given in Figure S8 (Supporting Information).

Modulation of material pore size has been shown to be an important parameter in modulating fouling characteristics of materials^[39]^ and their severity of the foreign body response^[40]^ along with ensuring suffcient mass transport and vascularization potential.^[41,42]^

### 2.3 Residual Cytotoxicity of PU-PEG Hydrogels: Influence of Catalyst and Casting Method

A preliminary high throughput screening of cytotoxicity revealed no significant impact on cell metabolic activity due to differences in hydrogel concentration, fabrication solvent, casting conditions, and nonstick coatings of the sheet casting materials (Figures S9 and S10, Supporting Information). There was, however, a significant impact seen on cells grown in media extracted from the DBTDL-catalyzed gels, even for the cells exposed to lower overall concentrations of the extracted media (Figure S11, Supporting Information), in line with previous reports in the literature of DBTDL cytotoxicity when the catalyst is mixed directly with cell culture media or if insuffcient DBTDL is extracted from polyurethane-amide materials.[] These trends were consistent regardless of the assay used (Figure S12, Supporting Information).

**Figure 3:**
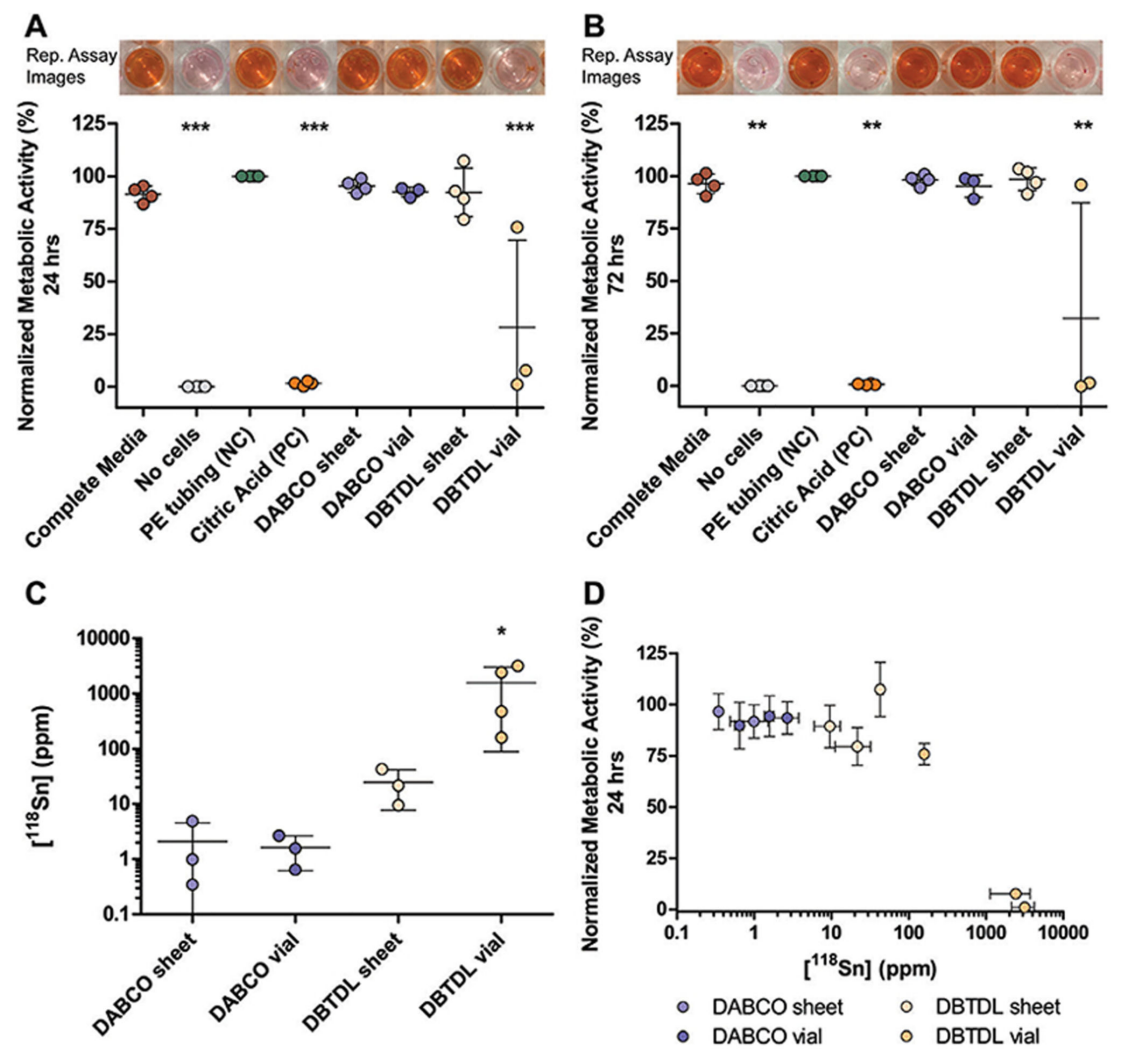
Catalyst substitution and sheet configuration casting condition removes cytotoxic effects Normalized metabolic activity of L929 fibroblast cells exposed to different media after A) 24 h and B) 72 h. Metabolic activity normalized to polyethylene tubing (green) for each experimental repeat. Internal controls where L929 cells were grown in their traditional complete media (red) and where no cells were included in the L929 traditional complete media (grey) were included for experimental rigor. Each data point represents the average normalized metabolic activity of cells grown in the presence of media extracted from 3–5 replicate gels from each of 3–4 independent syntheses (*n* = 3–4). Error bars represent standard deviation across the 3–4 independent syntheses. One-way ANOVA comparisons of conditions conducted with Tukey post-test, ***p<0.0001, **p<0.01 compared with PE tubing, complete media, DABCO sheet, DABCO vial, and DBTDL sheet samples. Representative assay samples in 96-well plate for each experimental group shown above their respective plots. C) ^118^Sn content in ppm of 23% (w/v) PU-PEG gels cast in the different configurations taken from the same syntheses used in cytotoxicity testing from A) and B). Each data point represents the average ^118^Sn content measured in 3 replicate gels from one of 3–4 independent synthesis (*n* = 3–4). Error bars depict standard deviation across 3–4 independent syntheses. Kruskal–Wallis Test with Dunn’s Multiple Comparison post-test, *p<0.05 with DABCO sheet. D) ^118^Sn content in ppm of 23% (w/v) PU-PEG hydrogels cast in the different configurations plotted against the normalized metabolic activity of L929 cells 24 h after treatment with extracted media from the respective hydrogel samples. Each data point represents the average ^118^Sn content and normalized metabolic activity for 3–5 replicate gels from a single synthesis. Error bars are standard deviation in ^118^Sn content (*x*-axis) and normalized metabolic activity (*y*-axis) across the 3–5 replicates run for each synthesis

Concentration matched 23% (w/v) DABCO- and DBTDL-catalyzed PU-PEG formulations cast in the vial and sheet configuration were then selected and their cytotoxicity was assessed according to the ISO 10993-5 standards. L929 fibroblasts grown in the presence of media extracted from 23% (w/v) DABCO-catalyzed PU-PEG hydrogels cast in sheet and vial configurations exhibited 95.40% ± 3.10% and 92.50% ± 2.36% metabolic activity, respectively. Gels cast using a concentration of DABCO three times greater did not reduce viability significantly (Figure S10, Supporting Information). Interestingly, cells grown in the presence of 23% (w/v) DBTDL-catalyzed PU-PEG hydrogels cast in a sheet configuration were also highly viable, exhibiting 92.25% ± 11.52% metabolic activity. However, cells that interacted with media extracted from 23% (w/v) DBTDL-catalyzed PU-PEG hydrogels cast in a vial configuration showed significantly reduced metabolic activity, 28.17% ± 41.39%, after 24 h (Figure 3A). The metabolic activity levels after 72-h exposure to media extracted from the various hydrogel formulations were similar to the 24h levels, with cells exhibiting normalized metabolic activities of 98.18% ± 2.69%, 95.17% ± 5.29%, 98.43% ± 5.46%, and 32.30% ± 55.00% when cultured in media extracted from sheet- and vial-cast 23% (w/v) DABCO-catalyzed PU-PEG hydrogels and sheet and vial-cast 23% (w/v) DBTDL-catalyzed PU-PEG hydrogels, respectively (Figure 3B).

The reduced metabolic activity seen by the cells exposed to the DBTDL-catalyzed gels cast in a vial set-up corresponded with the higher tin content detected in these samples by ICP-MS (Figure 3C, D), implying a higher retained amount of DBTDL in gels cast in this formulation. Interestingly, in one repeat of these experiments, the DBTDL-catalyzed vial cast gels did not exhibit as extreme of an impact on metabolic activity and the gels from these runs similarly seemed to contain a significantly lower concentration of tin as detected by ICP-MS (Figure 3D, dark yellow). From the matched ICP-MS and metabolic activity data, the threshold where tin concentrations impact cell metabolic activity appears to lie somewhere between 200 and 1000 ppm (Figure 3D). Casting these gels in a sheet configuration typically enabled more effective removal of DBTDL, with gels containing 24.70 ± 16.94 ppm of tin after washing, a level well tolerated by L929 cells. Media extracted from gels containing DBTDL was similar in pH to other samples, so we attribute the toxicity specifically to residual organotin in these samples (Figure S13, Supporting Information). Although levels of Sn(II) leaching from standard commercial polyurethanes are acceptably low, these findings demonstrate that considerations should be made around the replacement of toxic reagents and solvents when developing thicker porous elastic polyurethane-based co-polymer gel biomaterials, resembling the materials characterized herein, where leaching might be significant. Extraction of catalyst can be conducted effciently through thorough washing of thinner sheet-cast hydrogels, but catalyst may prove more diffcult to sufficiently extract from bulk materials.

### 2.4 Nonfouling Characteristics and Implant Tolerability of PU-PEG Hydrogels

Nonspecific binding of proteins or cells to surfaces can trigger the recruitment of a cascade of immune and inflammatory cells that play a role in the foreign body response and can encourage biomaterial failure and further tissue damage. The release of reactive oxygen intermediates and degradative enzymes from neutrophil degranulation or frustrated phagocytosis can damage implanted devices, such as polyurethane-based implants^[45]^. Fibrotic encapsulation can isolate implanted devices and disrupt tissue interactions, increasing impedance of implanted electrodes or creating the need for more frequent recalibration of blood glucose sensors.^[46,47]^ Materials which limit these initial adhesive events could therefore be of significant interest as implantable biomaterials. The nonfouling capacity of the various PU-PEG hydrogel formulations was assessed through the adsorption and infiltration of albumin and fibrinogen, the most common proteins in the blood serum, and the attachment of L929 mouse fibroblasts. Poly(dimethylsiloxane) (PDMS) was mechanically matched to the 23% (w/v) DABCO-catalyzed PU-PEG hydrogels and was included in the nonfouling experiments as a model common coating for implants or as an implantable material itself.^[48,49]^

**Figure 4:**
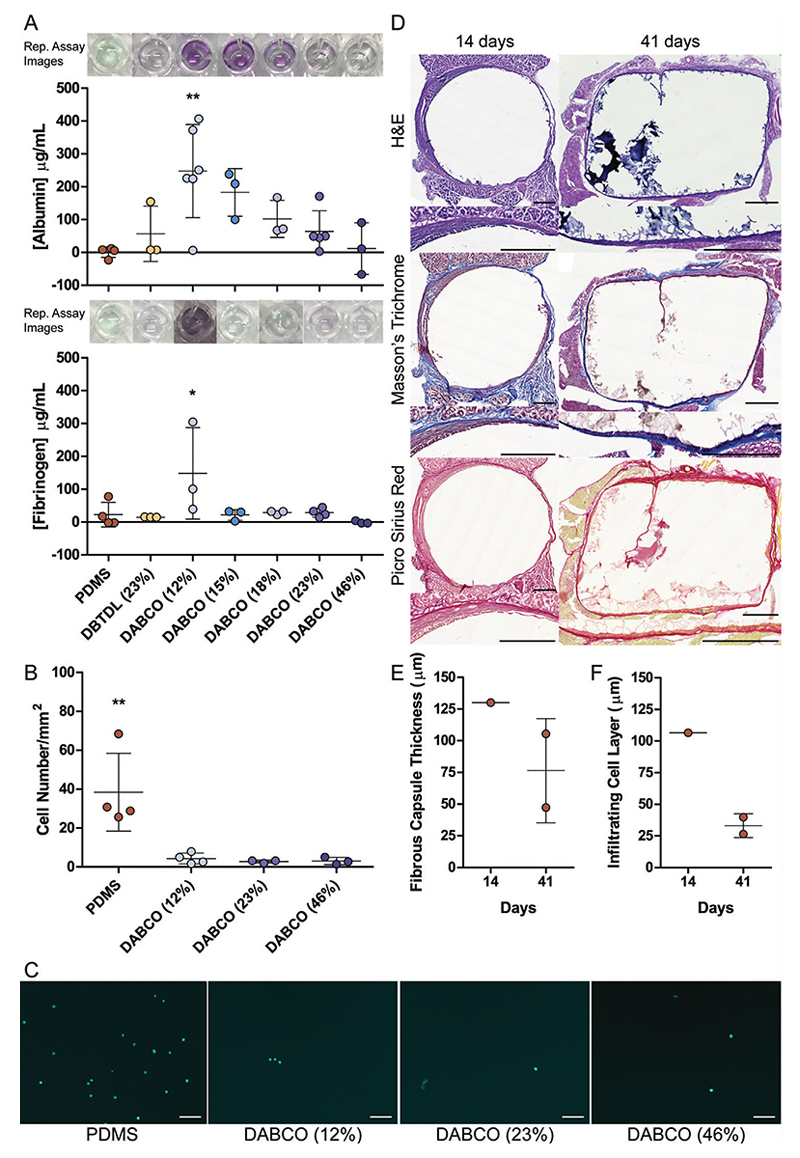
Nonfouling behavior and long-term tolerability of PU-PEG implants. A) Albumin (top panel) and fibrinogen (bottom panel) adsorption and infiltration behavior in 23% (w/v) DBTDL-catalyzed and 12% (w/v), 15% (w/v), 18% (w/v), 23% (w/v), and 46% (w/v) DABCO-catalyzed PU-PEG hydrogels compared with PDMS, assessed by bicinchoninic acid (BCA) assay. Each data point represents average albumin or fibrinogen adsorbed in a single experiment on at least 3 replicate gels, experiments repeated at least 3 times (*n* = 3–5). Representative images of albumin (top) and fibrinogen (bottom) adsorption assay samples for each formulation in 96-well plate shown above respective sample data in plots. B)Quantification of L929 fibroblast cell adhesion to 12% (w/v), 23% (w/v), and 46% (w/v) DABCO-catalyzed PU-PEG hydrogels compared with PDMS. Each data point represents average number of cells per mm^2^ measured in single experiment with at least 3 replicate gels, experiments repeated at least 3 times (*n* = 3–4). Overall average and standard deviation marked with a line and bars, respectively. Adsorption of albumin and fibrinogen and cell adhesion on each PU-PEG hydrogel formulation compared with PDMS by one-way ANOVA, Dunnett’s Multiple Comparison Test, *p<0.05, **p<0.005. C) Representative DAPI stained images of L929 fibroblast cells adhered to 12% (w/v), 23% (w/v), and 46% (w/v) DABCO-catalyzed compared with PDMS. Scale bars are 100 μm. D) H&E (top), Masson’s Trichrome (middle), and Picro Sirius Red (bottom) staining of 46% (w/v) DABCO catalyzed sheet cast PU-PEG implants and surrounding tissue 14 days (left) and 41 days (right) after implantation around vagus nerve. Scale bars are 500 μm. Quantification of E) the fibrous capsule and F) the infiltrating cell layer conducted on Masson’s Trichrome and Picro Sirius Red stainings each performed on 5–8 tissue sections from 1 and 2 mice for 14 and 41 day time points, respectively. The thickness of each layer was measured at 10 locations around the implant in each section of each staining. Each data point represents the overall average of the quantified layer determined by Masson’s Trichrome and Picro Sirius Red staining.

Interestingly, albumin adsorption and infiltration seemed to correlate with PU-PEG concentration, with the more concentrated gels exhibiting reduced protein adsorption and the more dilute gels displaying a higher degree of protein adsorption (Figure 4A, top). Adsorption of fibrinogen was indistinguishable for PDMS and PU-PEG formulations of 15% (w/v) and above, only the 12% (w/v) DABCO-catalyzed formulation displayed significant adsorption (Figure 4A, bottom).

The observed differences in protein adsorption levels could depend on the differences in protein size, which could impact surface adsorption and bulk infiltration;^[50,51]^ albumin is a 65 kDa globular protein,^[52]^ while fibrinogen is a larger (340 kDa) rodshaped serum protein.^[53]^ Alternatively, changes in surface hydrophobicity at different PU-PEG concentrations may contribute to the improved nonfouling ability at higher concentrations.^[54^–^57]^ Serum proteins typically adsorb more efficiently onto hydrophobic surfaces,^[54,58,59^,] but the greater charge density of albumin may allow it to bind to more hydrophilic surfaces than fibrinogen. Increased density of presented PEG chains is also widely implicated in reducing protein adsorption.^[60,61]^ The formation of a protein-resistant hydrated surface is thought to prevent non-covalent interactions between protein and gel. Previous literature suggests this effect is less pronounced for albumin than fibrinogen, consistent with our findings here.^[62]^ Given the observed median pore ranges of the various PU-PEG hydrogel formulations span 5.17 µm (IQR: 4.86 µm) to 26.10 µm (IQR: 35.92 µm) for 46% (w/v) to 12% (w/v) DABCO-catalyzed PU-PEG hydrogels, respectively (Figure 2F), it is likely that these proteins are able to infiltrate the PU-PEG hydrogel network in addition to their adsorption to the surface of the materials.

The extreme formulations, 12% (w/v) and 46% (w/v) DABCO-catalyzed PU-PEG hydrogels, exhibiting the full range of protein adsorption characteristics, along with mechanically matched 23% (w/v) DABCO-catalyzed PU-PEG hydrogels were then compared with PDMS gels in an adhesion assay with L929 mouse fibroblast cells. All three PU-PEG formulations exhibited similar low levels of cell adhesion (3.06 ± 1.83, 2.78 ± 0.74, 4.31 ± 2.79 cells mm^-2^) and were all statistically significantly below the 38.42 ± 20.07 cells mm^-2^ that adhered to the PDMS samples (Figure 4B, C).

The FBR is a major obstacle to the translation of biomaterials to the clinic, but nonfouling materials tend to exhibit a diminished FBR and good long-term implant tolerance.^[47]^ The 46% (w/v) DABCO-catalyzed PU-PEG hydrogel formulation exhibited the lowest amount of protein adsorption and infiltration and was therefore selected for implantation around a mouse vagus nerve, mimicking an electrode format, to examine long-term tolerability in vivo. Thin fibrous capsules, 130.0 µm after 2 weeks as well as 47.3 µm and 105.4 µm after 6 weeks, were measured around the PU-PEG gels (Figure 4D, E), indicative of a mild FBR and an acceptable capsule thickness for biomaterial implants. Our results were in line with previous reports of fibrous capsule thickness observed around pure PEG hydrogels at ≈110 µm, while PEG hydrogels functionalized with the cell adhesion peptide, RGD, displayed a fibrous capsule ≈80 µm thick 28 days after subcutaneous implantation in mice.^[63^–^65]^

## 3 Conclusions

In summary, we have developed a convenient one-pot strategy for preparing and casting cyto-compatible, nonfouling PU-PEG hydrogels with tunable mechanical and physical properties that suit a range of different applications in various tissue types. Our synthetic approach yields nontoxic hydrogels through the use of a less toxic organocatalyst, easily removeable greener solvents, and a facile sheet casting set-up that can be adapted for a range of applications. The stiffness, swelling behavior, and porosity of the gels can be varied simply by modulating their concentration, offering a convenient and reproducible means of matching gel properties to a wide range of biological tissues. The PU-PEG hydrogel formulations also demonstrate nonfouling characteristics and long-term stability. We anticipate that the simplified fabrication methodology and tunability of physicochemical properties developed here may encourage the use of PU-PEG hydrogel formulations in a broader range of biomaterials laboratories; and that the materials innovations demonstrated herein may act as a platform for tailored biomaterial implant technologies. We envisage that our methodology could possibly be readily adapted into commercial kits for use in laboratory and clinical settings. Further derivatization of the scaffolds to improve compatibility with a range of tissues is a key goal of future work in this area.

## 4 Experimental Section

### Materials

Anhydrous dimethylsulfoxide (DMSO) (99.9%, Product 276855), anhydrous dimethylformamide (DMF) (99.8%, Product 227056), anhydrous tetrahydrofuran (THF) (99.9%, inhibitor-free, Product 401757), anhydrous acetonitrile (ACN) (99.8%, Product 271004), 10K polyethylene glycol (10K PEG) (BioUltra, Product 92897-F, Lot BCCB7356), (1,4-diazabicyclo[2.2.2]octane) (DABCO) (99%, D27802, Lot WXBC2274V), and 1,6-diisocyanatohexane (HMI) (99%, Product 52649, Lot BCBZ8006) were purchased from Sigma-Aldrich. di-*n*-butyltin dilaurate (DBTDL) (>95%, Product 71130, Lot W24B0211H), 1H,2H,2H-perfluorooctyltrichlorosilane (FOTS) (96%, Product L16606, Lot 101218683) was purchased from Alfa Aesar. 1,1,1-tris(hydroxymethyl)ethane (TME) (97%, Product 824324, lot R27C029) and microscope slides (Product 631-1553) were purchased from VWR. Western Blot Mini-Protean slides were purchased from BioRad (Product 1653308). Silicone gaskets were fashioned from high-temperature silicone rubber sheets from MacMaster-Carr (Products 3788T21, 3788T22).

### Slide Silanization

Glass slides (either Western Blot or microscope slides) were functionalized with FOTS by vapor deposition. Briefly, slides were washed sequentially with ultrapure water (Milli-Q Q-Pod, Merck) and ethanol and thedried at 65 °C for 24 h, before placing carefully in a desiccator with FOTS (200 µL). A vacuum was applied for 15 min, then the desiccator was sealed and kept under a static vacuum for a further 45 min.

### General Procedure for PU-PEG Synthesis

Solid reagents (10K PEG, DABCO, and TME) were dried in a desiccator for 24 h under vacuum prior to use, using phosphorus pentoxide as desiccant. All glassware, stirrer bars and septa were dried at 65 °C for 24 h prior to use. A 24 mL glass vial was charged with 10K PEG, TME and catalyst (either DABCO or DBTDL) at a molar ratio of 1.0:0.7:0.05. A magnetic stirrer bar was added and the vial sealed using a rubber septum under a continuous flow of nitrogen gas. Anhydrous solvent (DMF, DMSO, THF, ACN) was added such that the final mass/volume percent (w/v) of the hydrogels was of a known value between 9.2 and 223% (Table S2, Supporting Information). The mixture was stirred and heated to dissolution using a heat gun. HMI (5 equivalents with respect to 10K PEG) was added via syringe and the mixture stirred for a further 10 min before transfer via syringe to the desired casting mold. The solution was then cured at 85 °Cfor24hinanoven.

After 24 h, gels were removed from the oven and retrieved from the curing setup. Gels were washed by submerging in stirred THF (24 h), then ultrapure water (72 h; Milli-Q Q-Pod, Merck) to remove unreacted material and organic solvents.

### Vial Configuration

Gels were prepared following the above procedure, with curing taking place in the reaction vial.

### Silicone Gasket Manufacture

Silicone gaskets were cut from high-temperature silicone rubber sheets (0.5–1.5 mm high, MacMaster-Carr, Product 3788T21, 3788T22) with a scalpel to the appropriate outer dimensions of the microscope slides (dimensions 26 × 76 mm) or western blot glasses (dimensions 101 × 74 mm) with a border diameter of 5 or 9 mm, respectively.

### Western Blot Sheet Configuration

Western Blot curing setups were prepared using either untreated or FOTS-functionalized slides (see above). Slides (dimensions 101 × 74 mm) and silicone gaskets (9 mm diameter, 0.5–1.5 mm height) were dried at 65 °C for 24 h prior to assembly. After drying, the gaskets were sandwiched between two slides and pressed tightly with binder clips to ensure an air-tight seal. The apparatus was flushed with nitrogen gas for 3 min before use.

The synthetic procedure was carried out as described above, but the reaction mixture (≈4–5 mL) was transferred via syringe to the Western Blot casting mold prior to curing. To facilitate injection, a bleed needle was inserted into one side of the apparatus (Figure 1D) and the reaction mixture injected into the other side.

### Microscope Slide Sheet Configuration

Microscope slide curing setups were prepared using either untreated or FOTS-functionalized slides (see above). Slides (dimensions 26 × 76 mm) and silicone gaskets (5 mm diameter, 0.5–1.5 mm height) were dried at 65 °C for 24 h prior to assembly. After drying, the gaskets were sandwiched between two slides and pressed tightly with binder clips to ensure an air-tight seal. The apparatus was flushed with nitrogen gas for 3 min before use.

The synthetic procedure was carried out as described above, but the reaction mixture (≈1-1.5 mL) was transferred via syringe to the microscope slide casting mold prior to curing.

### Reaction Monitoring by Infrared Spectroscopy

Infrared spectra were recorded using a Cary 630 FTIR spectrometer (Agilent Technologies) with single bound diamond ATR. Measurements were made between 4000 and 650 cm^–1^ at a resolution of 8 cm^–1^, 200 scans per data point.

PU-PEG hydrogels were prepared in the vial setup as described above (DABCO catalyst), at concentrations of 12%, 23%, and 46% (w/v). At known time points between 0 and 24 h, 100 µL aliquots of the reaction mixture were taken and IR spectra immediately recorded. At time points following the onset of gelation, extraction via syringe was not possible, and instead small portions of the gel were removed at each time point by spatula for analysis. Spectra of hydrogel samples following the THF and H^2^O washing steps were also recorded.

### Rheology

After water washes, PU-PEG hydrogels were trimmed, if necessary, and cut with an 8 mm hollow punch (BOEHM). Characterization of material mechanical properties were performed with AR2000ex dynamic shear rheometer (TA Instruments) with 8 mm diameter parallel plate geometry. All rheological sweeps were conducted at 22.5 °C. Oscillatory frequency sweeps of 1–100 rad s^-1^ were conducted at a fixed strain amplitude of 0.01 (1%) strain. Oscillatory strain sweeps from 0.002 to 1 (0.2–100%) strain were completed at an angular frequency of 10 rad s^-1^. The average of at least 3 hydrogel replicates was collected for a single data point. The presented results are the mean values collected from at least 3 independent syntheses.

### Swelling Ratio and Degradation Studies

PU-PEG hydrogels cast in the sheet configuration were cut with a 10 mm punch (BOEHM). Hydrogel degradation profiles were assessed based on methods outlined in Speidel et al.^[66]^ Wet masses of hydrogels were determined after ultrapure water (Milli-Q Q-Pod, Merck) washes following synthesis (*m*_iw_) and samples were distributed into 5 groups for each time point (0, 1, 7, 14, 35 days) so that there was no statistically significant difference in the starting wet masses across the groups. One group of 3 samples for each formulation were lyophilized (FreeZone 2.5 L Benchtop Freeze Dryer, LabConco) to identify the initial dry mass (m_id_). Remaining samples were placed into histology cassettes and placed into a beaker of at least 800 mL of ultrapure water (Milli-Q Q-Pod, Merck) and incubated at 37 °C (Isotemp GPD 28 water bath, Fisher Scientific). At each time point up to 35 days, one group of 3 samples for each formulation was blotted dry and the swollen masses (*m*_s_) of the hydrogel samples wer measured. The hydrogels were lyophilized overnight (FreeZone 2.5 L Benchtop Freeze Dryer, LabConco) and the dry masses of the hydrogel samples (m_d_) were collected. Representative gels for each formulation and time point were imaged whenever a mass was collected.

The initial dry mass (*m*_id_calc_) for each hydrogel was calculated by multiplying miw of individual hydrogels by the average of the initial dry masses divided by the initial wet mass of the samples assessed immediately after fabrication (*m*_id_/m_iw_).

The % mass loss at each time point is calculated as

(1) $$\%\text{mass}\;\text{loss}=\frac{m_{id\_calc}-m_{d}}{m_{id\_calc}}\times 100$$

The mass swelling ratio, *q*, was determined at each time point as

(2) $$q=\frac{m_{s}}{m_{d}}$$

### SEmml

For investigation of ethanol wash impact on PU-PEG gel surfaces, 4 mm punches (BOEHM) of the sheet configuration 15% (w/v), 18% (w/v), and 46% (w/v) DABCO-catalyzed PU-PEG gels were cut, immersed, and washed in absolute ethanol (Fisher Scientific, E/0600DF/17, Lot: 2066678) twice for at least 30 min on a roller mixer (Stuart SRT6D, BioCote). Samples were then immersed and washed in ultrapure water (Milli-Q Q-Pod, Merck) six times for at least 30 min on a roller mixer (Stuart SRT6D, BioCote).

Ethanol washed samples, control 4 mm punches of 15% (w/v), 18% (w/v), and 46% (w/v) DABCO-catalyzed PU-PEG sheet samples were directly lyophilized overnight (FreeZone 2.5 L Benchtop Freeze Dryer, Lab-Conco) for the assessment of the impact of ethanol washes (Figure S8). Vial-cast 23% (w/v) DBTDL-catalyzed, 12% (w/v), 15% (w/v), 18% (w/v), 23% (w/v), and 46% (w/v) DABCO-catalyzed PU-PEG gels were dipped into liquid nitrogen and freeze-fractured. Samples were then lyophilized overnight (FreeZone 2.5 L Benchtop Freeze Dryer, LabConco). Samples were sputter-coated (Qourum 150T ES sputter coater) with 10 nm platinum and imaged on a Zeiss Gemini Ultra 55 scanning electron microscope with an on-axis SE (InLens SE) and Everhart-Thornley SE (SE2) detectors.

Feret diameter distributions were determined from at least 86 traced total representative pores collected from at least 3 representative images of each vial-cast formulation in ImageJ software. The median and interquartile range (IQR) of the distributions were determined in Microsoft Excel (Equation (3)).

(3) |QR=Q3−Q1

### Cell Culture

The NCTC clone 929 (L929, CCL-1) cell line of mouse fibroblasts (ATCC) was cultured in complete media composed of ATCC-formulated Eagle’s Minimum Essential Medium (EMEM, Catalog No. 30-2003) with 10% (v/v) fetal bovine serum (FBS) (Sigma, Product No. BCBV7611) and 100 U mL^-1^ penicillin, 100 µg mL^-1^ streptomycin (Gibco, Ref: 15140-122) at37 °C, 5% CO_2_. L929 cells were passaged at 80% confluence every 2 or 3 days by trypsinization with TrypLE (Gibco, Ref: 12604013). L929 cells for all experiments were used at passage 10–12.

### ISO Standard 10993-5 Cytotoxicity Assay

PU-PEG hydrogel formulations and polyethylene tubing (PE20, Becton Dickinson and Company, #427406, Lot #: 5019565) were trimmed into pieces no larger than 3 mm in any dimension and washed twice for at least 1 h in absolute ethanol (Fisher Chemical, Product Code: E/0650DF/17) and an additional 6 times for at least 1 h in autoclaved ultrapure water (Milli-Q Q-Pod, Merck) on a roller mixer (Stuart SRT6D, BioCote) at room temperature. After removal of excess water from the final wash, hydrogel materials and polyethylene tubing were lyophilized (Labconco, Kansas City, MO) at least overnight and EMEM was added at a base ratio of 1 g dry gel mass per 5 mL complete media, adjusted for the individual swelling ratio of each hydrogel formulation. Citric acid (Sigma Aldrich, Product No. 251275-100G) positive control solution was prepared at 8.484 mg mL^-1^ EMEM. Extraction solutions were placed in a shaking incubator (New Brunswick S41i, Eppen-dorf)for24hat37°C, 125 RPM. Passage 10 L929 cells were seeded at a density of 20000 cells cm^-2^ into clear 96-well plates (Sarstedt, 83.3924) for the standard assay. After 24 h in shaking incubator, extracted media from each of the PU-PEG formulations and controls was collected through vacuum filtration through Steriflip (0.2 µm) tube top filtration units (Merck Millipore, Cat No. SCGP00525) or through syringe collection (Henke Sass Wolf, Ref: 4050-00010) and filtration (UNIFLO 0.2 µm, Whatman, Cat No. 9916-1302, Lot #: 190815-425) and FBS and penicillin/streptomycin was added to final concentrations of 10% (v/v), 100 U mL^-1^, 100 µg mL^-1^, respectively. 100 µL of complete extracted media for each condition was then added to 3–5 wells for each condition. After 24- or 72-h incubation at 37 °C, 5% CO_2_, 10 µL cell counting kit-8 reagent (Sigma-Aldrich, Product No. 96992-500TESTS-F) was added to each well and incubated for 3 h for the 24 h conditions and 2 h for the 72 h conditions. Absorbance was then read at 450 nm on a Varioskan Lux microplate reader and SkanIt Software 4.0 (Thermo Fisher Scientific). Each reading was collected 3 times. Experiments were repeated at least 3 times with hydrogel samples prepared from independent syntheses.

### ISO Standard 10993-5 High Throughput Cytotoxicity Assay

Extraction solutions were prepared in the same manner described in the standard assay. In the high throughput experiments, Passage 10 L929 cells were seeded (Viaflo 384, INTEGRA) at a density of 5000 cells cm^-2^ in white 384-well plates (Corning, Product No. 3765) for Cell-Titer Glo assay (Promega, Product No. G7571) and black 384-well plates (BD Falcon, Product No. 353962) for Hoechst (stock solution 2 × 10^-3^ m in H_2_O; Sigma-Aldrich, Product No. 14533) cell counting and cultured for 24 h at 37 °C, 5% CO_2_. Master mixes of extracted media solutions from each material treatment at dilutions of 100% (v/v), 50% (v/v), 25% (v/v), and 10% (v/v) with complete media were prepared on a MANTIS liquid handler (FORMULATRIX) into V-bottom 384-well plates (Corning). Cells were treated with 30 µLof master solutions with 3–5 wells for each condition. After 24 and 72 h incubation at 37 °C, 5% CO_2_, plates for Hoechst staining were stained with 7% (v/v) Formaldehyde (Sigma Aldrich, Product No. F8775), 7 × 10_-6_ m Hoechst solution (Sigma Aldrich, Product No. 14533) with a MultiFlo dispenser (Echo550, Labcyte) in the dark at room temperature for 20 min. Plates were washed with PBS twice (HydroSpeed, Tecan) and left in 40 µL PBS for imaging. Images were acquired using the IN Cell Analyzer 2200 (GE Healthcare) with a 4× objective. Quantitative image analysis was run in CellProfiler (http://www.cellprofiler.org/). Representative analysis pipeline displayed in Figure S14 (Supporting Information).

CellTiter-Glo Luminescent Cell Viability Assay (Promega, Product No. G7571) was prepared according to manufacturer’s instructions and diluted 1:4 in PBS (1×). 25 µL of CellTiter-Glo reagent was added to each well with a MultiFlo dispenser (Echo550, Labcyte) and shaken for 2 min at 450 rpm (Titramax 1000, Heidolph). Plates were incubated in the dark at room temperature for 10 min and then luminescence readings were taken on Infinite M200PRO plate reader (Tecan), 0.1 s well^-1^. Analysis of imaging and CellTiter-Glo data was performed using KNIME software (https://www.knime.com/blog/a-workflow-for-high-throughput-screening-data-analysis-processing-and-hit-identification). Representative analysis pipeline displayed in Figure S15 (Supporting Information).

### ICP-MS

Samples for Inductively Coupled Plasma-Mass Spectrometry (ICP-MS) were prepared by digesting a known mass (3–40 mg) of dried solid hydrogel in a mixture of hydrochloric acid (1.00 mL; 37 wt% in H_2_O, 99.999% trace metals basis) and nitric acid (100 µL; 70%, purified by redistillation, ≥99.999% trace metals basis). After 2 days at room temperature, the acidic solutions were homogenized then diluted with ultrapure water by a known factor (100–1000×) to fall within the range of the ICP-MS Sn calibration curve. ICP-MS measurements were performed on a PerkinElmer Nexion 300X mass spectrometer operating in standard mode with 1 s integration time for all elements measured (^118^Sn, ^103^Rh, ^192^Ir). A calibration curve for 200 ppt to 200 ppb Sn was used for quantitation. A 10 ppb Rh/Ir internal standard solution was added into the flow system and used to correct for any variation in plasma energy. Nebulizer gas flow was optimized on the day with the daily performance tune solution. Argon Plasma gas flow 16 L min^-1^, ICP power 1.5 kW. Samples were injected with a cetac ASX520 autosampler.

### Albumin and Fibrinogen Adsorption Quantification

PDMS (Sylgard 184 Elastomer Kit) for nonfouling experiments was prepared at a ratio of 50 parts base to one part curing agent, degassed, and cured at 65 °C for 48 h to mechanically match the 23% (w/v) PU-PEG formulation^[48,49]^. 6 mm punched (BOEHM) PU-PEG disks of each formulation and PDMS were prepared and incubated in 1 mg mL^-1^ bovine serum albumin (BSA; Sigma, Product No. A2153-100G, Lot #: SLBX5725) or 4 mg mL^-1^ fibrinogen (EMD Millipore, Product No. 341573-1GM, Lot #: 3612283) in PBS solution (1×, Gibco, Product No. 18912-014, Lot #: 1746484) for 2.5 h at room temperature. Disks were then washed three times in fresh PBS for 1 min each, to wash away excess BSA or fibrinogen. Calibration curve solutions for known concentrations of both albumin and fibrinogen were prepared in the presence of 23% (w/v) DABCO-catalyzed PU-PEG hydrogel samples (Figure S16, Supporting Information). Retained protein was then quantified through the BCA Protein assay (Thermo Fisher Scientific), prepared according to manufacturer’s instructions, where sample absorbance was measured at 562 nm on a microplate reader (Thermo Fisher Scientific, Product No. 23227).

### Nonfouling Cell Adhesion Studies

6 mm punched (BOEHM) PU-PEG disks of each formulation and PDMS were washed twice for at least 1 h in absolute ethanol (Fisher Chemical) and an additional 5 times for at least 1 h in autoclaved ultrapure water (Milli-Q Q-Pod, Merck) on a roller mixer (Stuart SRT6D, BioCote) at room temperature. Gels were then placed in plates and incubated in 200 µL of L929 complete media for 30 min at 37 °C, twice. L929 cells were then seeded at 15000 cells cm^−2^ and allowed to attach for 24 h at 37 °C, 5% CO_2_. Samples were washed gently 3 times in PBS to remove unattached cells and then fixed for 15 min in 4% (v/v) paraformaldehyde (Histolab, Product No. 02176). Samples were washed again 3 times in PBS and then stained with 14.3 × 10^−3^ m DAPI (Life Technologies, Product No. D1306) in 1% (w/v) BSA in PBS. Samples were washed again 3 ti in PBS to remove excess DAPI and then placed on coverslips and the number of cells per sample was counted on the Axio Imager.M2 (Zeiss). Representative images of each sample were collected.

### Mouse Implant Model

3 mm punched (BOEHM) 46% (w/v) PU-PEG disks were washed for at least 30 min 4 times in 70% ethanol and 4 times in autoclaved ultrapure water (Milli-Q Q-Pod, Merck) for at least 30 min on rollers and stained with sterile-filtered (Acrodisc 0.2 µm syringe filter, Henke Sass Wolf Fine-Ject 21 G needles, Norm-Ject 10 mL Leur lock) blue food coloring (Dr. Oetker) overnight. Male C57BL6J mice (age 8–10 weeks, Charles River laboratories) were housed on 12-h light and dark cycle at 25 °C with ad libitum access to food and water. Anesthesia was induced with isoflurane and a 1:1 mixture of oxygen and air. Mice were placed in supine position and a ventral midline cervical incision was performed between the mandible and sternum. Subcutaneous tissues were moved laterally to expose the salivary glands, which were then gently separated to reveal the right cervical vagus nerve (Figure S17B, Supporting Information). A scalpel incision was made from the circumference to the center of 3 mm 46% (w/v) PU-PEG disks and a 27 G needle was used to open the center to a suffcient diameter to fit around the vagus nerve (Figure S17A, Supporting Information) and the disk was placed around the right vagus nerve and the severed edge of the disk was sutured together to secure the disk in place around the nerve (Figure S17C, Supporting Information). The salivary glands and subcutaneous tissue were gently moved back into place and the mice were sutured closed. Mice recovered on a heating pad until they regained appropriate righting reflexes and were then returned to their cages. After 14 and 41 days the mice were euthanized under CO_2_ anesthesia and perfused with sequential intracardial injections of PBS and 4% (v/v) paraformaldehyde in phosphate buffered saline (PBS). All animal work was completed according to the ethical treatment guidelines for animals given by the Stockholm Regional Board for Animal Ethics (Stockholm, Sweden, N104/16, 20818-2020).

### Histology and Immunohistochemistry

After perfusion with PFA, mouse heads were collected and covered in 4% (v/v) paraformaldehyde for at least 2 days at 4 °C. Tissue was then moved either into 30% (w/v) sucrose solution or optimal cutting temperature compound (OCT) for at least 5 days at 4 °C. The material and surrounding issue was then dissected out and placed into optimal cutting temperature compound (OCT) overnight at4 °C. The samples were then washed with OCT and embedded in cryomolds (Tissue-Tek, Sakura Finetek, Netherlands), frozen on dry ice. 10 µm sections were collected on a cryostat and mounted on microscope slides (Superfrost, Thermo Fisher Scientific).

### H&E Staining

Sections were brought to room temperature for 15 min under the hood. After sections were hydrated in PBS (1×, Gibco, Product No. 18912-014), they were stained with Mayer’s hematoxylin solution, Lillie’s modification (Dako, Ref: S3309, Lot #: 10148347) for 3 min. Sections were washed in tap water at room temperature until color no longer leached from the slides, and then were washed in ultrapure water (Milli-Q Q-Pod, Merck). Bluing reagent (Dako, Ref: CS702, Lot #: 072297) was added to the sections for 2 min and then washed with ultrapure water (Milli-Q Q-Pod, Merck). Sections were then stained with 1% (w/v) Eosin Y solution for 1 min before being washed with ultrapure water (Milli-Q Q-Pod, Merck) until no further color leached from the sections. Sections were dehydrated in a series of 70% (v/v) (VWR, 83801.360), 90% (v/v), and 100% (v/v) ethanol (Fisher Scientific, 10048291), a minute in each. Sections were cleared in xylenes (Sigma-Aldrich, Product No. 534056-500 mL) 3 times for 5 min and then mounted in Diamount (Diapath, Ref: 030400, Lot: 2016XIII26) and sealed with clear nail polish (DIAMANT, #3501). Sections were imaged with the Axio Scan.Z1 Digital Slide Scanner (Zeiss).

### Masson’s Trichrome

Sections were brought to room temperature for 15 min under the hood. After sections were hydrated in PBS (1×, Gibco, Product No. 18912-014), they were stained with the Masson’s Trichrome kit (DiaPath, Ref: 010210, Lot: 2020×17501) according to manufacturer’s instructions. Sections were dehydrated in a series of 70% (v/v) (VWR, 83801.360), 90% (v/v), and 100% (v/v) ethanol (Fisher Scientific, 10048291), a minute in each. Sections were cleared in xylenes (Sigma-Aldrich, Product No. 534056-500 mL) 3 times for 5 min and then mounted in Diamount (Diapath, Ref: 030400, Lot: 2016XIII26) and sealed with clear nail polish (DIAMANT, #3501). Sections were imaged with the Axio Scan.Z1 Digital Slide Scanner (Zeiss).

### Picro Sirius Red

Sections were brought to room temperature for 15 min under the hood. After sections were hydrated in PBS (1×, Gibco, Product No. 18912-014), they were stained with the Picro Sirius red stain kit (Abcam, ab150681, Lot #: GR3363951) according to manufacturer’s instructions. Sections were rinsed with 100% (v/v) ethanol (Fisher Scientific, 10048291), three times for one minute. Sections were cleared in xylenes (Sigma-Aldrich, Product No. 534056—500 mL) 3 times for 5 min and then mounted in Diamount (Diapath, Ref: 03 0400, Lot: 2016XIII26) and sealed with clear nail polish (DIAMANT, #3501). Sections were imaged with the Axio Scan.Z1 Digital Slide Scanner (Zeiss).

### Fibrous Capsule Assessment

The thickness of the fibrous capsule and infiltrating cell region were both measured in 10 different locations around the material on at least 5 sections stained with Masson’s Trichrome and Picro Sirius Red using the ZEN lite (Zeiss) software.

### Statistical Analysis

All results from at least 3 independent experiments were statistically analyzed and a summary of the statistical tests implemented and the significant differences found are provided in the relevant figure captions. Any pre-processing steps are outlined in the caption. Unless otherwise noted, the results were displayed as mean and standard deviation. Appropriate statistical tests were selected according to the distribution of the individual data set. All statistical analysis was conducted in PRISM 5.

### Ethics Approval Statement

All animal experiments were conducted according to ethical treatment guidelines for animals given by the Stockholm Regional Board for Animal Ethics (Stockholm, Sweden, N104/16, 208182020).

### Supporting Information

Supporting Information is available from the Wiley Online Library or from the author.

## Supplementary Material

Supplementary materials

## Data Availability

The data that support the findings of this study are available from the corresponding author upon reasonable request.
