# Supplementary material for: Tailored Biocompatible Polyurethane-Poly(ethylene glycol) Hydrogels as a Versatile Nonfouling Biomaterial
Source: Adv Healthc Mater. Author manuscript; Available in PMC 2024 Jan 5. (PMC7615486; doi:10.1002/adhm.202201378)
Supplement: Supplementary materials [file EMS156636-supplement-Supplementary_materials.pdf]

ADVANCED HEALTHCARE MATERIALS

Supporting Information

for *Adv. Healthcare Mater.*, DOI 10.1002/adhm.202201378

Tailored Biocompatible Polyurethane-Poly(ethylene glycol) Hydrogels as a Versatile Nonfouling Biomaterial

*Alessondra T. Speidel, Phillip R. A. Chivers, Christopher S. Wood, Derrick A. Roberts, Inês P. Correia, April S. Caravaca, Yu Kiu Victor Chan, Catherine S. Hansel, Johannes Heimgärtner, Eliane Müller, Jill Ziesmer, Georgios A. Sotiriou, Peder S. Olofsson and Molly M. Stevens**

Supporting Information

Tailored Biocompatible Polyurethane-Poly(ethylene glycol) Hydrogels as a Versatile Non-fouling Biomaterial

*Alessondra T. Speidel, Phillip R. A. Chivers, Christopher S. Wood, Derrick A. Roberts, Inês P. Correia, April S. Caravaca, Yu Kiu Victor Chan, Catherine S. Hansel, Johannes Heimgärtner, Eliane Müller, Jill Ziesmer, Georgios A. Sotiriou, Peder S. Olofsson, Molly M. Stevens**

Table S1. Solvent compatibility with PU-PEG synthesis.

Solvent	Gelation
THF	No
DMF	Yes
DMSO	Yes
ACN	Yes

Table S2. Selected panel of PU-PEG hydrogel concentrations % (w/v) and their standard synthesis component ratios.
Note: *for each synthesis only one catalyst (either DABCO or DBTDL) is used in the reaction mixture*

Calculated Mass/Volume Percent (%)	Solvent Volume (mL)	PEG Mass (mg)	HMI Volume (µL)	TME Mass (mg)	DABCO Mass (mg)	DBTDL Volume (µL)
12% (w/v)	19.4	2000	220	33.5	1.57	-
15% (w/v)	14.6	2000	220	33.5	1.57	-
18% (w/v)	12.1	2000	220	33.5	1.57	-
23% (w/v)	9.7	2000	220	33.5	1.57	220
46 % (w/v)	4.9	2000	220	33.5	1.57	-

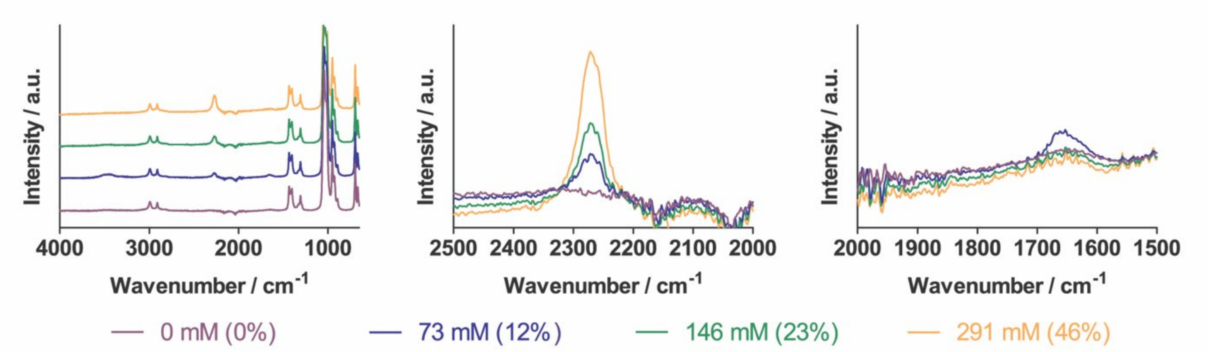

Figure S1. IR spectra of HMI dissolved in DMSO. Full spectrum (left) and spectra expanded in regions for isocyanate (middle) and carbonyl (right) peaks.

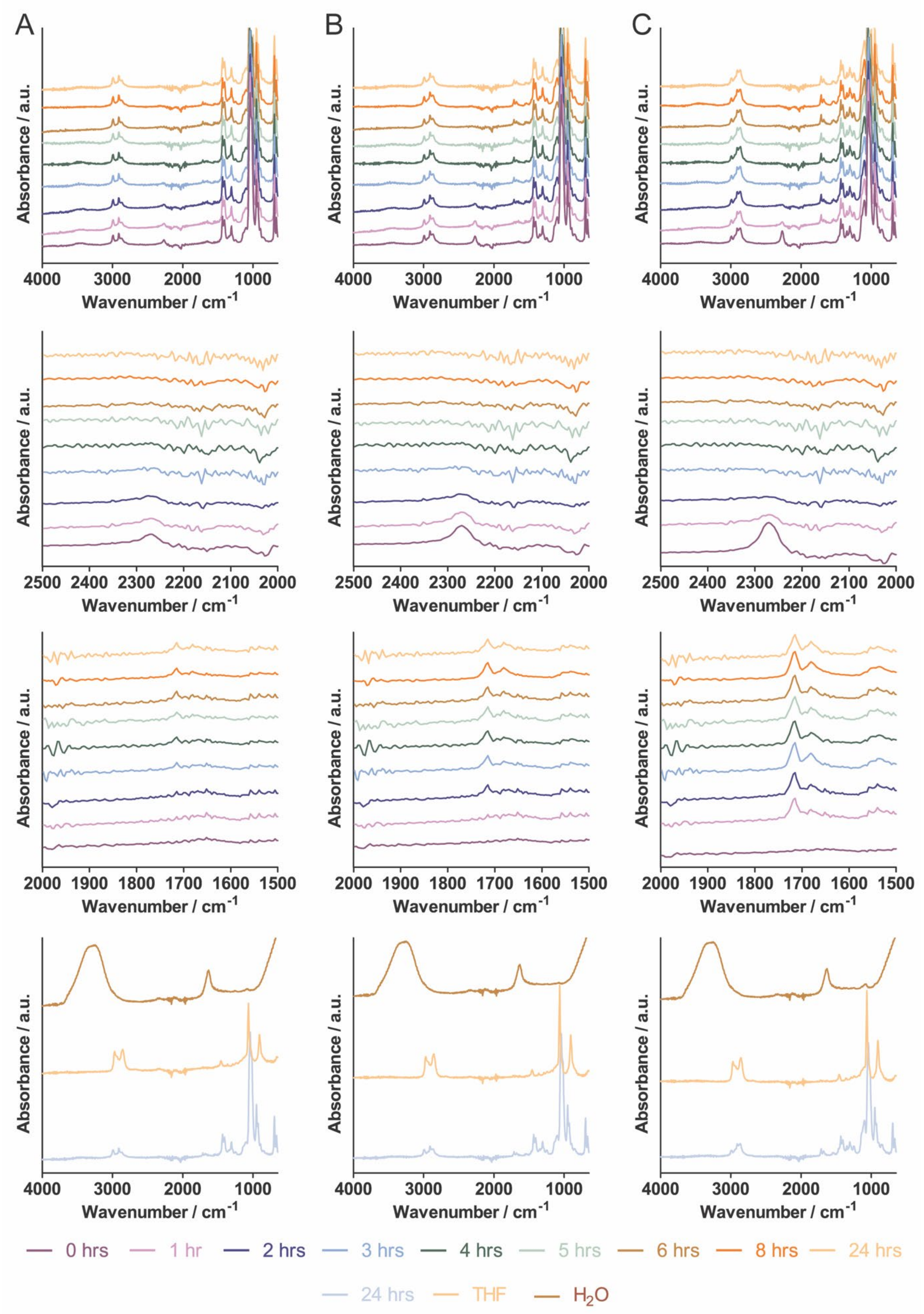

Figure S2. IR spectroscopic data for PU-PEG curing reactions at (A) 12%; (B) 23%; and (C) 46% (w/v) reactant concentration. Spectra are shown for time points between 0 hrs (defined as the time the sample was placed in the curing oven) and 24 hrs. Top row: Full spectra (4000-

800 cm^{-1}); Second row: Expanded isocyanate region; Third row: Expanded carbonyl region; Fourth row: Full spectra after fabrication (light blue) and then after THF (gold) and DI H_2O (brown) washing steps.

The synthesis of the PU-PEG hydrogels was unsuccessful in THF, but was successful in DMSO, DMF and ACN solvents (Table S1) and this variation in solvent did not have significant impact on the mechanical, swelling, or degradation properties of the resulting hydrogel materials (Figures S3). The relationship between mechanical properties, G*, and concentration of the hydrogel formulations was maintained independent of the solvent the gels were cast in (Figure S3A,B). Successful syntheses were observed over a broad range of concentrations spanning 9.2% (w/v) to 223% (w/v) PU-PEG generating hydrogels with complex shear moduli ranging over 2 orders of magnitude from ~800 Pa (823 ± 195 Pa) to ~190000 Pa (189867 Pa), nearly spanning the dynamic range of mechanical behavior exhibited by soft brain tissue (300-400 Pa) to stiffer cartilage tissue (0.1 – 2.5 MPa).[1,2]

Swelling ratios were similar for hydrogels of the same concentration, independent of the solvent the gels were cast in (Figure S3C). Mass loss over 14 days for the various formulations was low (Figure S3D). An inverse linear relationship between the swelling ratio and mechanical properties of the hydrogels was maintained independent of the solvent the hydrogels were cast in (Figure S3E).

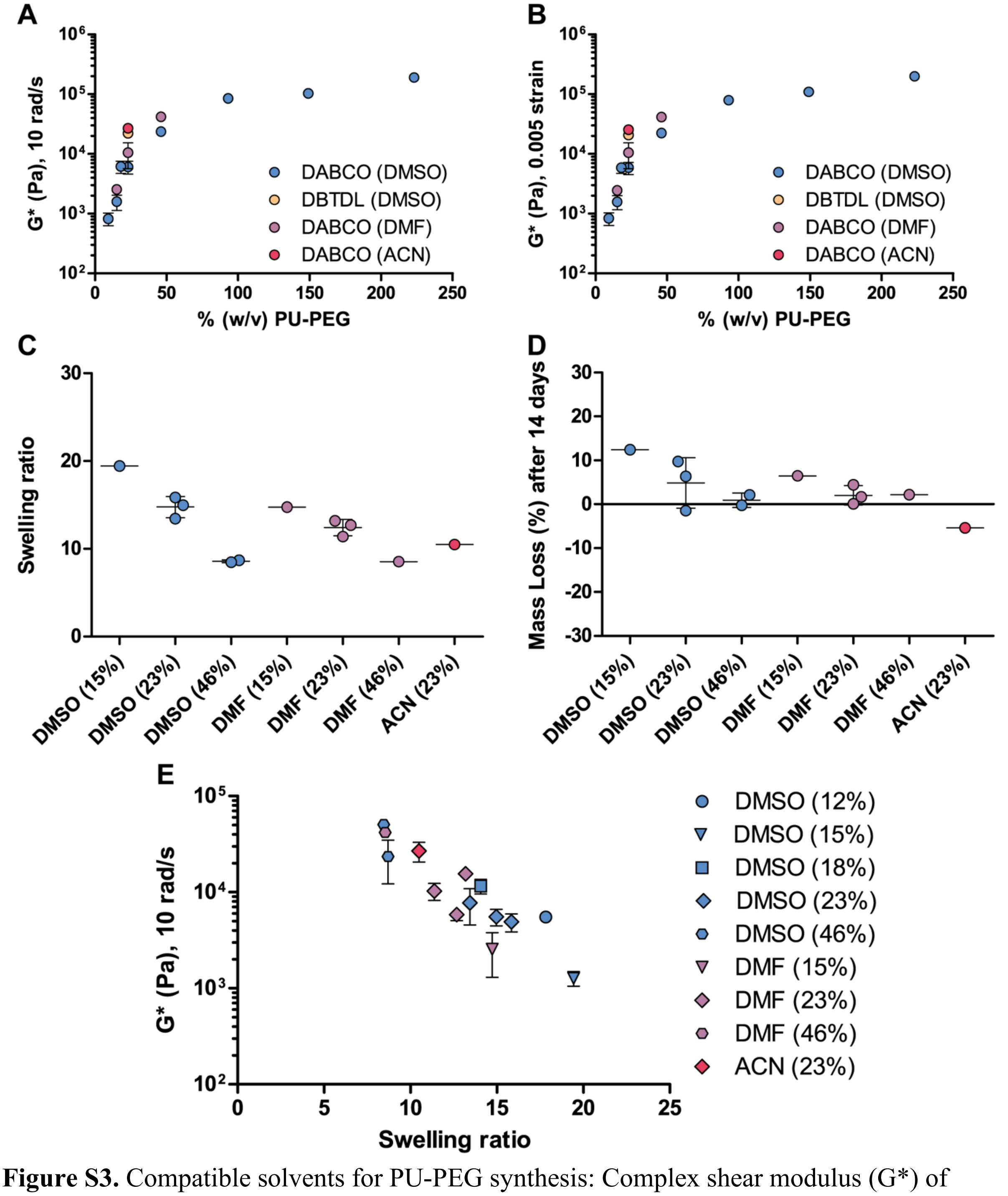

Figure S3. Compatible solvents for PU-PEG synthesis: Complex shear modulus (G*) of varied % (w/v) concentrations of PU-PEG DABCO-catalyzed hydrogels cast in DMSO (blue), DMF (purple), and acetonitrile (pink) and DBTDL-catalyzed hydrogels cast in DMSO (gold) at (A) 10 rad/s and (B) 0.005 strain. (C) Swelling ratio and (D) mass loss after 14 days of 46% (w/v), 23% (w/v), 15% (w/v) PU-PEG DABCO-catalyzed hydrogels cast in DMSO (blue) and DMF (purple) and 23% (w/v) PU-PEG DABCO-catalyzed cast in acetonitrile (pink) in 37 °C water. (E) Complex shear modulus (G*) as a function of swelling ratio (q) for the DABCO-catalyzed hydrogels at various concentrations cast in DMSO (blue), DMF (purple), and acetonitrile (pink). Each data point represents the average of 3 samples run from a single synthesis. If samples from more than two syntheses were included an error bar representing the standard deviation is displayed.

The mechanical performances of the various formulations of PU-PEG hydrogels were compared in oscillatory frequency sweeps (Figure S4) and oscillatory strain sweeps (Figure S5). Figure S4 displays the complex shear modulus (G*) from representative samples of each fabrication of each hydrogel formulation across 1-100 rad/s at a fixed strain amplitude of 0.01 (1%) strain and 22.5 °C. The evaluated frequency sweep ranges incorporate physiologically relevant frequencies, such as those associated with body movements, walking ~1 – 8 Hz (6.28 – 50.27 rad/s) to running ~3-100 Hz (18.85 – 628 rad/s),[3] average breathing rate ~0.2 – 0.3 Hz (1.26 – 1.88 rad/s), and average human heart rate: ~1 Hz (6.28 rad/s). A fixed strain amplitude of 0.01 strain was selected as all hydrogel formulations displayed behavior within the linear region until 0.02-0.03 strain (Figure S5). G* values for the various formulations were consistent across the range of frequencies surveyed, implying that the hydrogels exhibit consistent mechanical properties across the physiologically relevant frequency range investigated.

Figure S5 gives representative oscillatory strain sweeps for each synthesis of each hydrogel formulation from 0.002-1 (0.2-100%) strain at a fixed angular frequency of 10 rad/s and 22.5 °C. G* values remain consistent until 0.02-0.03 strain for all formulations.

Representative sweeps shown in dark red and light blue were each conducted on PU-PEG samples from independent batches synthesized by separate researchers and with different batches of reagents. A relative relationship between the different concentrations of gels and their complex shear moduli were maintained within the syntheses conducted by each independent researcher, but there was some variation in the overall mechanical properties between gels of the same formulation between the two. Establishment of relative calibration curves to account for variation in protocol interpretation, reagent variability, set-up humidity,

and other variation in laboratory environment and set-up are recommended. Each sweep is representative of runs conducted on at least 3 samples from each synthesis that was analyzed.

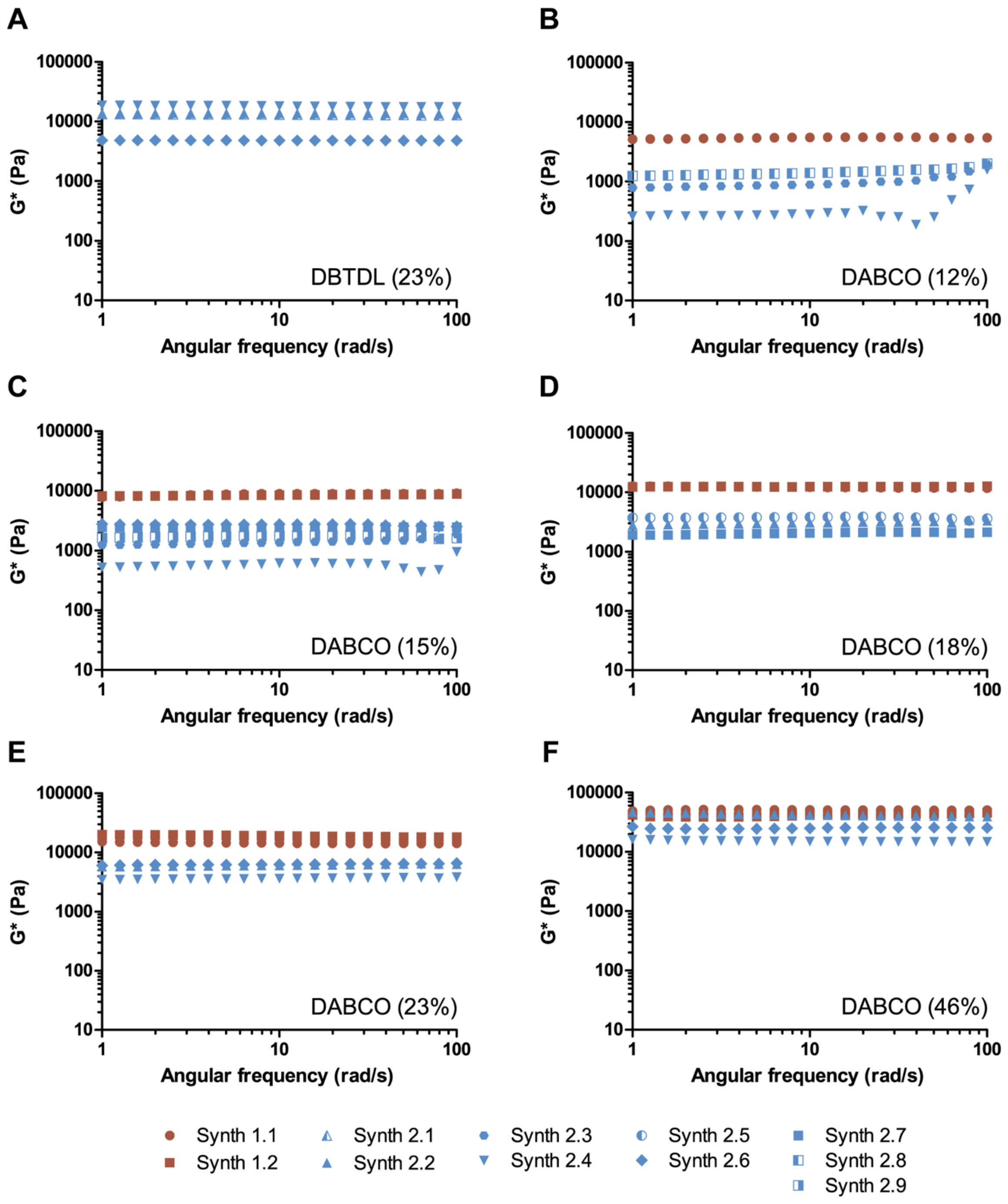

Figure S4. Representative frequency sweeps: Complex shear modulus (G*) of (A) 23% (w/v) DBTDL-catalyzed (B) 12% (w/v), (C) 15% (w/v), (D) 18% (w/v), (E) 23% (w/v), and (F) 46% (w/v) DABCO-catalyzed PU-PEG hydrogels at 22.5 °C 1-100 rad/s. Each sweep representative of at least 3 samples (overall average behavior) run from that synthesis. Dark red (synth 1) and light blue (synth 2) syntheses were fabricated by independent researchers. The first digit of each synthesis represents the researcher and the second digit indicates the repeat synthesis.

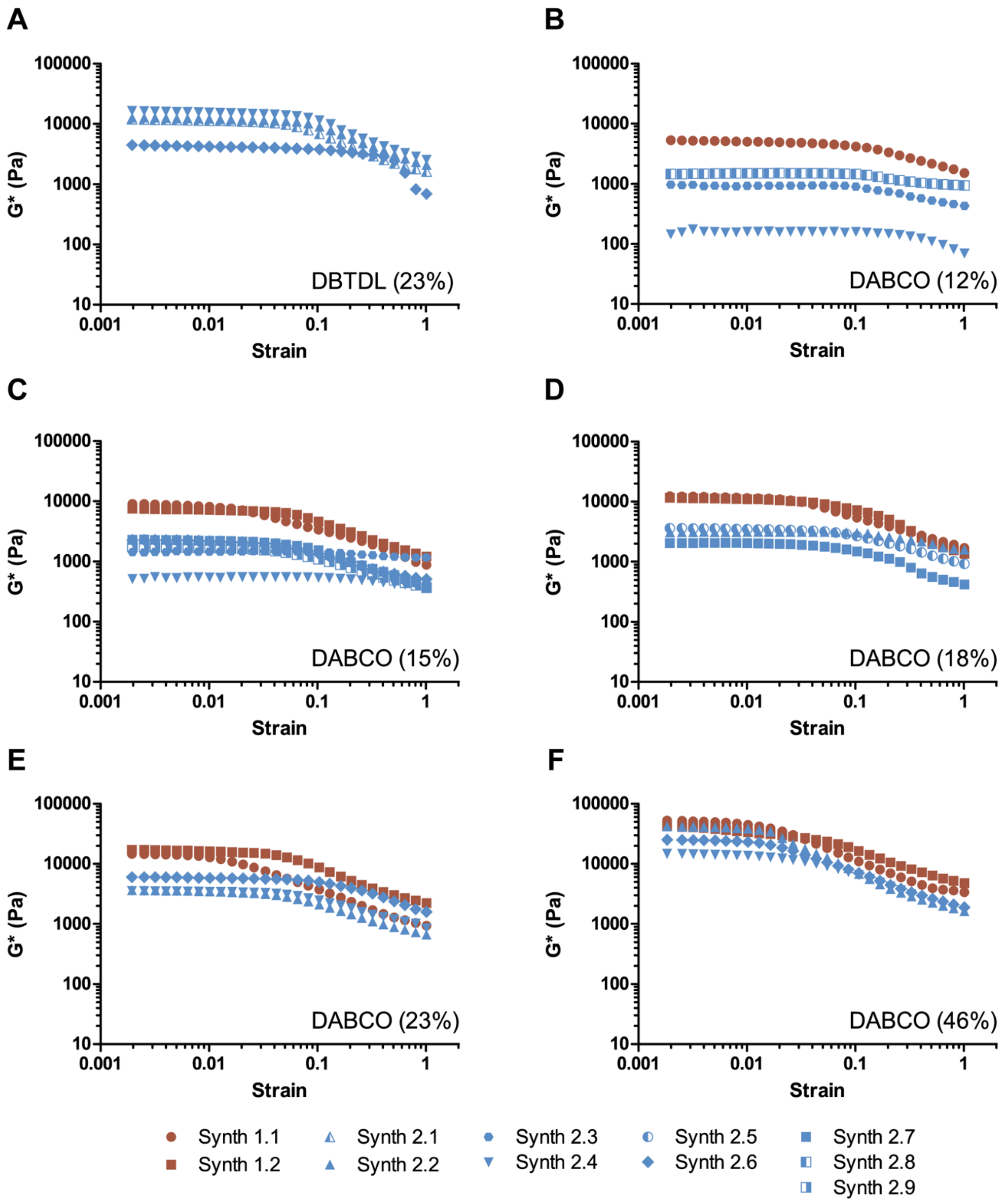

Figure S5. Representative Strain Sweeps: Complex shear modulus (G*) of (A) 23% (w/v) DBTDL-catalyzed (B) 12% (w/v), (C) 15% (w/v), (D) 18% (w/v), (E) 23% (w/v), and (F) 46% (w/v) DABCO-catalyzed PU-PEG hydrogels at 22.5 °C; 0.002-1 strain. Each sweep is representative of at least 3 samples collected (median sweep) and run from a single synthesis. Dark red (synth 1) and light blue (synth 2) syntheses were fabricated by independent researchers. The first digit of each synthesis represents the researcher and the second digit indicates the repeat synthesis.

The overall swelling profiles of the various hydrogel formulations were examined in ultrapure water maintained at 37 °C over 35 days (Figure S6) to establish an expectation of the

maximum swelling ratio exhibited by the various PU-PEG formulations. There was no statistically significant variation in the swelling ratios for any of the formulations across the 35-day period, representative images of swollen gels at each of the time points are displayed above the data for their respective time points. Each data point is the average swelling ratio for a single synthesis, determined from values exhibited by 3 samples collected and measured from each synthesis. The average of the swelling ratios determined for the 3 independent syntheses along with the standard deviations are depicted as lines in the plots below.

The PU-PEG gels' swelling ratios were assayed following the THF and ultrapure water washes at the end of the PU-PEG hydrogel synthesis. Following the ultrapure water washes in their synthesis, the final PU-PEG hydrogels were observed to swell 78-95% from their original cast area (data not shown). 46% (w/v), 23% (w/v), and 15% (w/v) PU-PEG hydrogels displayed a 76.5%, 126.8%, and 132.6% gain in mass between original synthetic mix and final hydrogels after the final ultrapure water wash (data not shown).

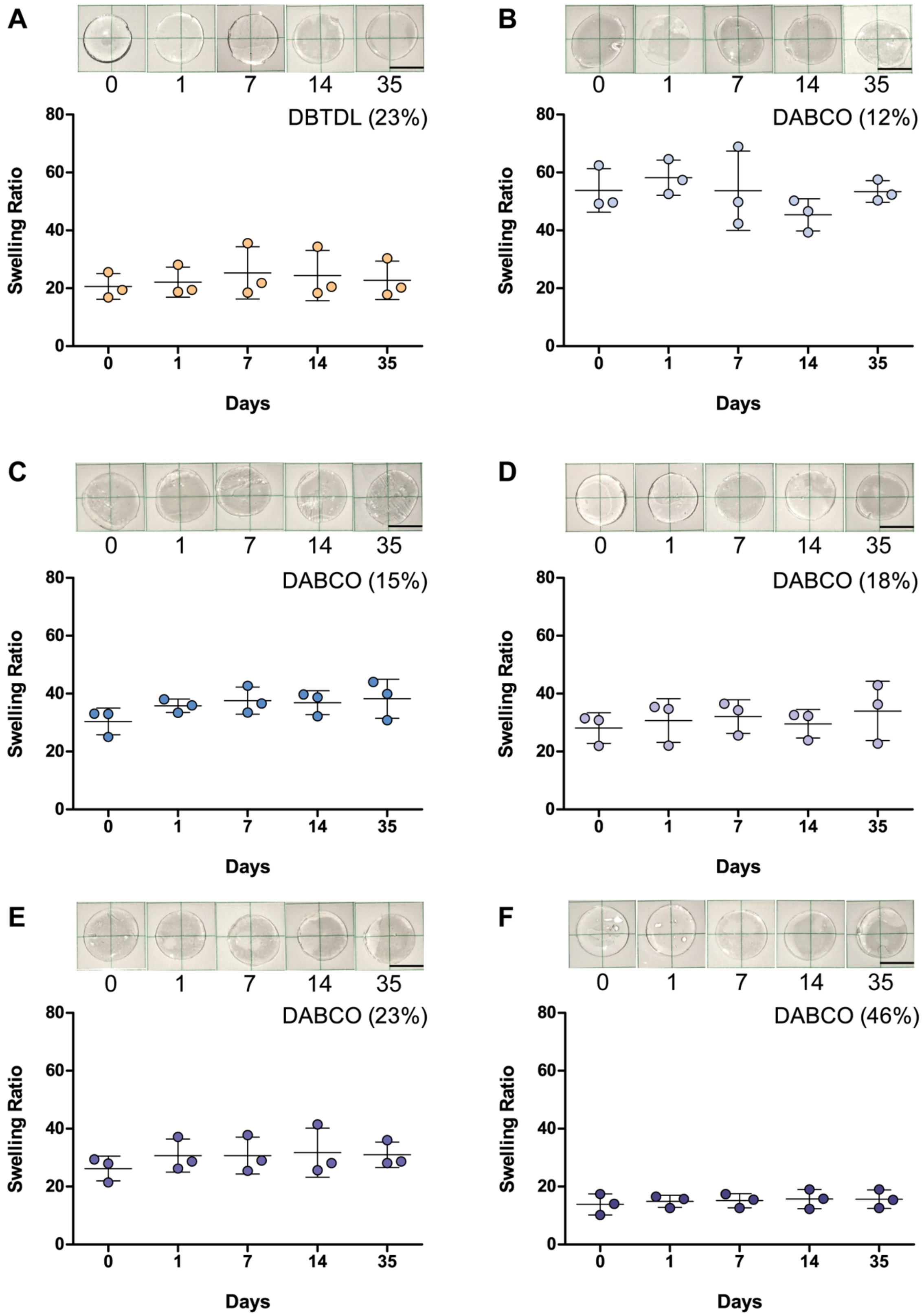

Figure S6. Swelling ratio over 35 days: Swelling ratio (q) of (A) 23% (w/v) DBTDL-catalyzed, (B) 12% (w/v), (C) 15% (w/v), (D) 18% (w/v), (E) 23% (w/v), and (F) 46% (w/v) DABCO-catalyzed PU-PEG hydrogels after 0 (after washes from synthesis), 1, 7, 14, and 35 days incubation in 37 °C water. Representative images of each gel formulation are shown above their respective time point and plot. Each data point represents the average of 3 samples run from a single synthesis. At least 3 independent syntheses were analyzed for each hydrogel

formulation. Samples compared by one-way ANOVA, Tukey post-test. No statistically significant differences detected. Scale bar is 5 mm.

The overall degradation profiles of the various hydrogel formulations were examined in ultrapure water maintained at 37 °C over 35 days (Figure S7) to establish the minimum expected degradation of the various PU-PEG formulations. There was no statistically significant variation in the mass loss determined for any of the formulations across the 35-day period, representative images of the lyophilized gels at each of the time points are displayed above the data for their respective time points. Each data point is the average mass loss for a single synthesis, determined from values exhibited by 3 samples collected and measured from each synthesis. The average mass loss determined for the 3 syntheses along with the standard deviations are marked under the data points in the plots below.

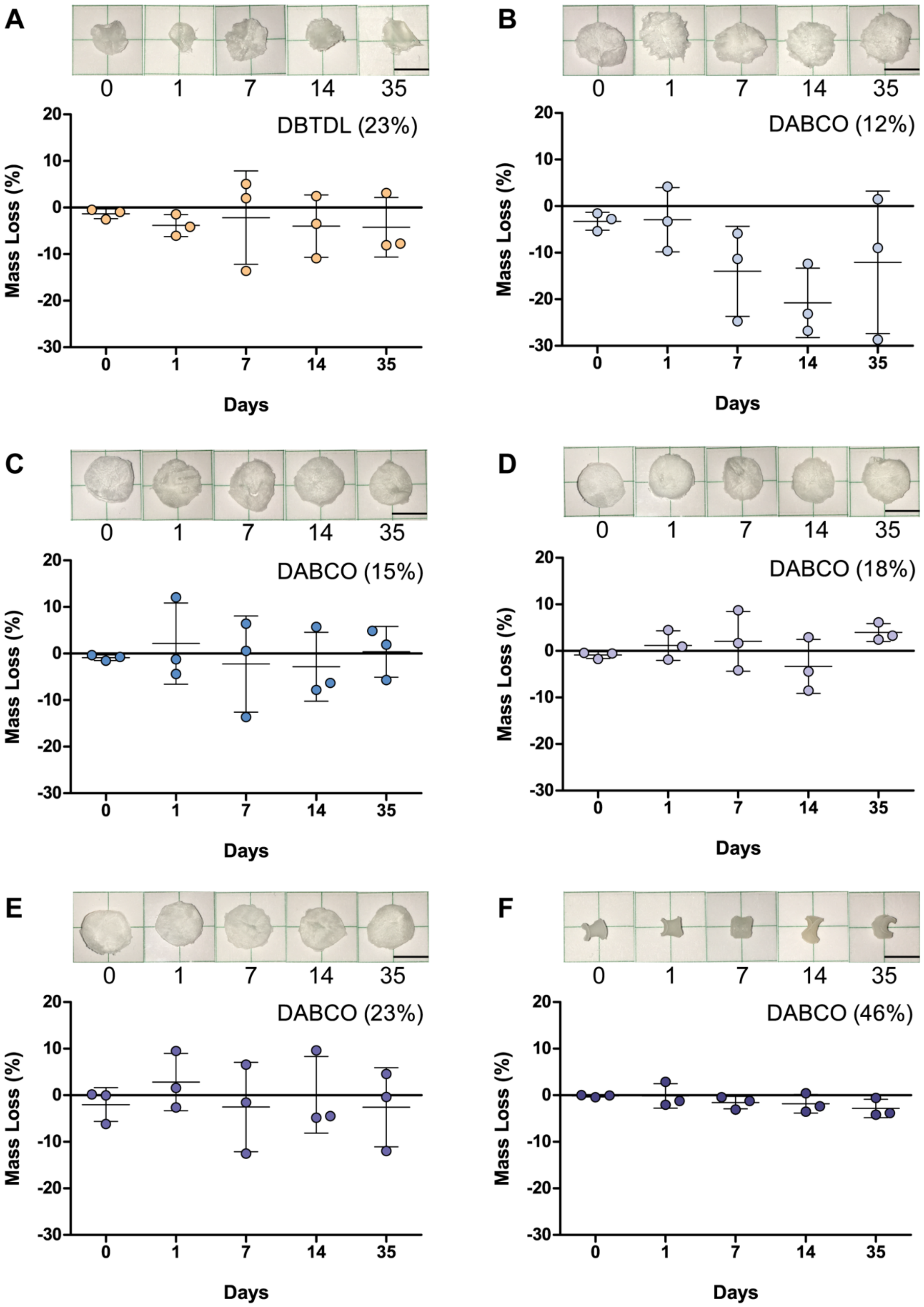

Figure S7. Mass loss over 35 days: Mass loss of (A) 23% (w/v) DBTDL-catalyzed, (B) 12% (w/v), (C) 15% (w/v), (D) 18% (w/v), (E) 23% (w/v), and (F) 46% (w/v) DABCO-catalyzed PU-PEG hydrogels after 0 (after washes from synthesis), 1, 7, 14, and 35 days incubation in 37 °C water. Representative images of each gel formulation are shown above their respective time point and plot. Each data point represents the average of 3 samples run from a single synthesis. At least 3 independent syntheses were analyzed for each hydrogel formulation.

Samples compared by one-way ANOVA, Tukey post-test. No statistically significant differences detected. Scale bar is 5 mm.

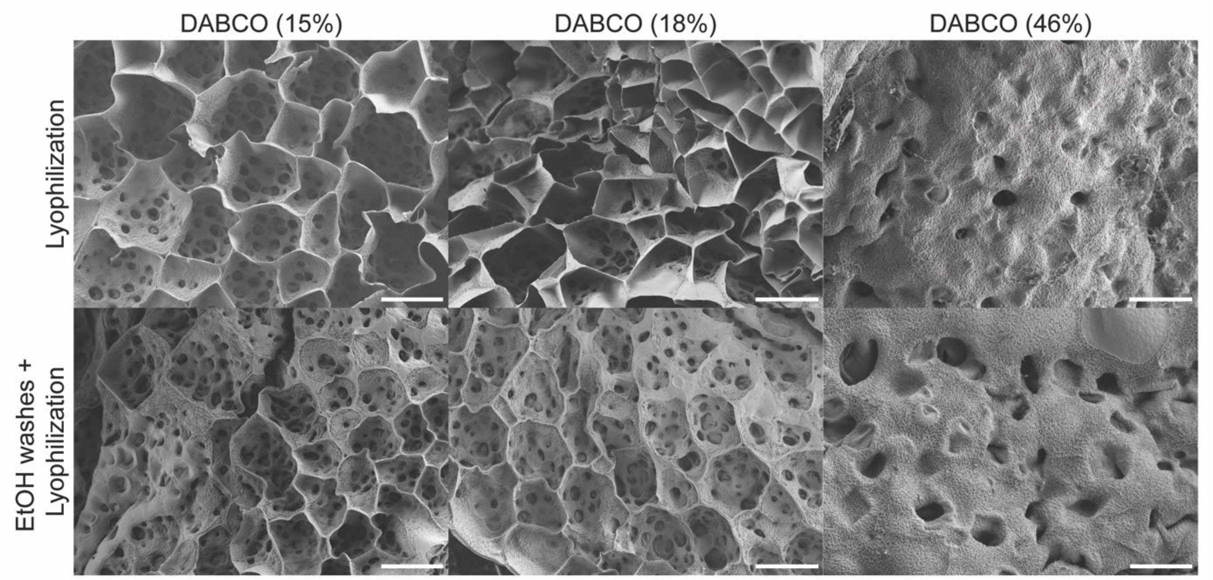

Figure S8. PU-PEG structure maintained with ethanol washes. SEM images displaying the pore structure of the surface structure of 3 mm cylindrical punches of 15% (w/v) (left), 18% (w/v) (middle) and 46% (w/v) (right) PU-PEG hydrogels prepared in the sheet configuration either directly lyophilized (top row) or washed with 100% (v/v) ethanol for at least one hr before six washes with water and lyophilized (bottom row). Scale bar is 50 µm.

PU-PEG gels cast in the various vial and sheet formats with and without FOTS and TMSCl coating agents were examined to ensure that these variations did not significantly impact the mechanical properties or introduce cytotoxic effects into the materials (Figure S9). The mechanical properties and coatings of the sheet set-up with FOTS and TMSCl agents were assessed rheologically and did not have a significant impact on the complex shear modulus displayed by the materials of the different preparations (Figure S9A,B). As an example, 18% (w/v) PU-PEG gels are shown to demonstrate this consistency in their resulting mechanical properties.

Metabolic activity of L929 fibroblast cells was not significantly impacted when treated with media extracted from FOTS-coated gels or gels cast with a higher concentration of DABCO over 24 or 72 hrs (Figures S9 and S10). Cells treated with media extracted from 18% (w/v) PU-PEG sheet-cast hydrogels uncoated, FOTS-coated, and FOTS-coated with 3× the amount

of DABCO had metabolic activities of 94.0%, 100.4%, and 97.9% after 24 hrs, respectively. Cells treated with media extracted from 15% (w/v) PU-PEG vial-cast and sheet-cast, FOTS-coated hydrogels exhibited metabolic activities of 105.8% and 88.87% after 24 hrs, respectively (Figure S9C,10C). Cells treated with media extracted from 18% (w/v) PU-PEG sheet-cast hydrogels uncoated, FOTS-coated, and FOTS-coated with 3× the amount of DABCO had metabolic activities of 96.71%, 97.09%, and 93.08% after 72 hrs, respectively. Cells treated with media extracted from 15% (w/v) polyurethane-PEG vial-cast and sheet-cast, FOTS-coated hydrogels exhibited metabolic activities of 99.85% and 92.26% after 72 hrs, respectively (Figure S9D, 10D). Each data point is the average of at least 3 replicates taken from a single synthesis. The standard deviations are marked for formulations where data was collected from gels from multiple syntheses.

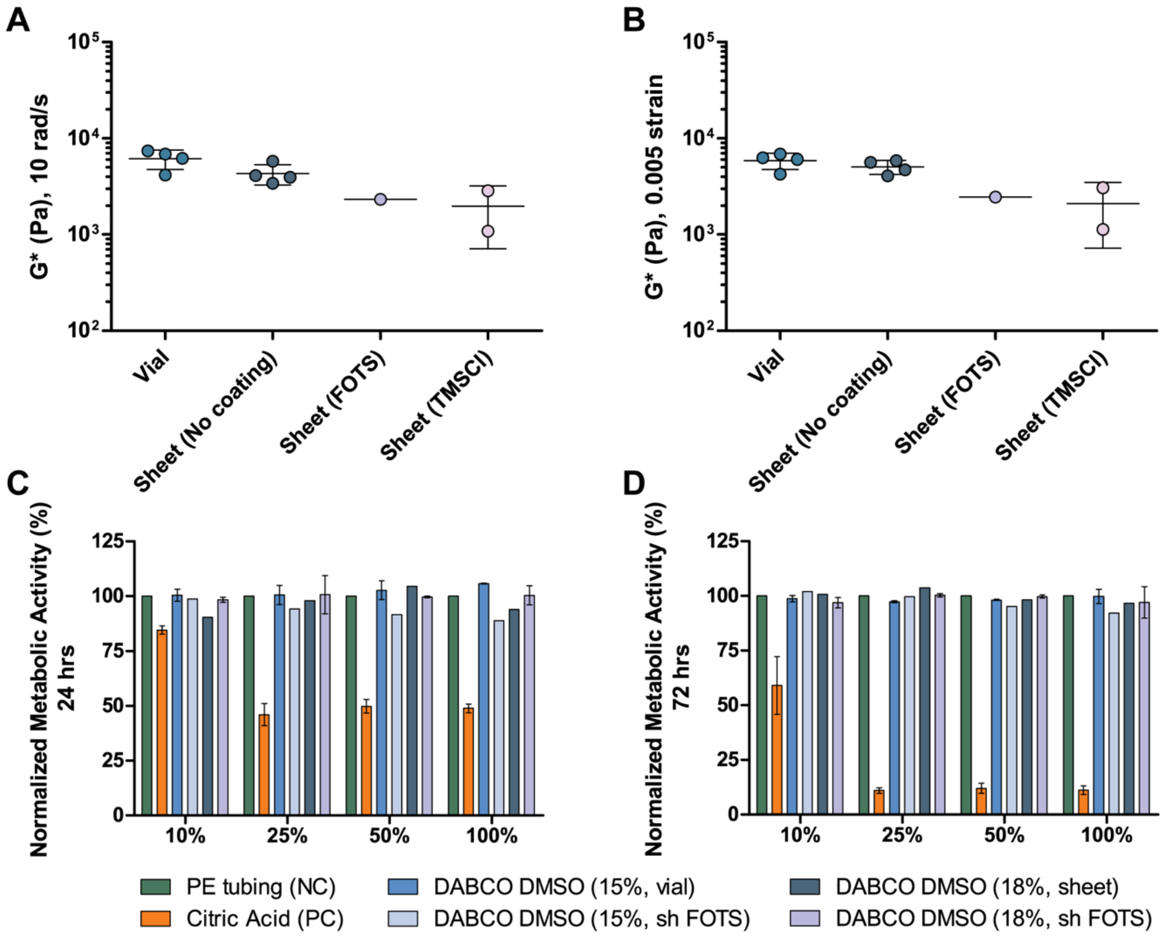

Figure S9. Hydrogel casting conditions and cytotoxicity: Complex shear modulus (G*) of 18% (w/v) PU-PEG DABCO-catalyzed hydrogels cast in vial (dark slate), uncoated sheet

(light slate), sheet with FOTS coating (periwinkle), and sheet with TMSCl coating (light pink) at (A) 10 rad/s and (B) 0.005 strain. Normalized metabolic activity of L929 fibroblasts exposed to media treated with varying concentrations of polyethylene tubing (green), citric acid (orange), 18% (w/v) DABCO-catalyzed sheet-cast (dark slate), 18% (w/v) DABCO-catalyzed FOTS-coated sheet-cast (light slate), 15% (w/v) DABCO-catalyzed vial-cast (blue) and 15% (w/v) DABCO-catalyzed FOTS-coated sheet-cast (light blue) after (C) 24 hrs and (D) 72 hrs. Each data point represents the average of 2-5 samples run from a single synthesis. If samples from more than two syntheses were included an error bar representing the standard deviation is displayed.

Increasing the concentration of DABCO catalyst does not significantly impact the mechanical properties or cytotoxicity of the hydrogel materials (Figure S10). Rheologically, 18% (w/v) PU-PEG hydrogels with 3 mM DABCO catalyst exhibit similar complex shear moduli to gels cast within 1 mM DABCO catalyst (Figure S10A,B). All rheology data is the average of 3 gel samples collected from a single synthesis. All metabolic activity data is the average of 2-5 replicate samples taken from a single run of the experiment.

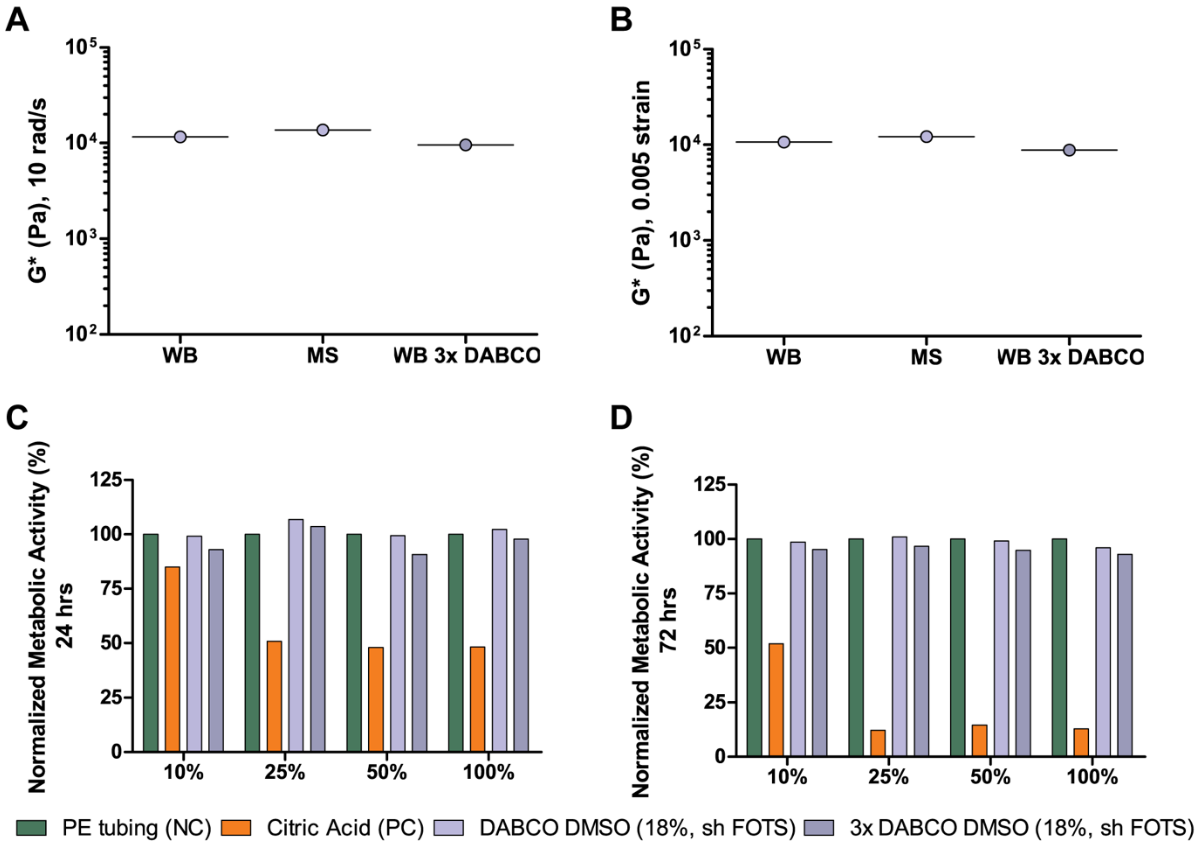

Figure S10. Hydrogel sheet casting conditions, DABCO concentration, and cytotoxicity: Complex shear modulus (G*) of 18% (w/v) PU-PEG DABCO-catalyzed hydrogels cast in FOTS-coated sheet (WB), microscope slide (MS), and sheet configurations with 3× DABCO concentration at (A) 10 rad/s and (B) 0.005 strain. Each data point represents the average of 3 samples run from a single synthesis. Normalized metabolic activity of L929 fibroblasts

exposed to media treated with varying concentrations of polyethylene tubing (green), citric acid (orange), 18% (w/v) DABCO-catalyzed FOTS-coated sheet-cast (light slate), 18% (w/v) 3× DABCO-catalyzed FOTS-coated sheet-cast (grey) after (C) 24 hrs and (D) 72 hrs. Each bar represents the average of 2-5 samples run from a single synthesis.

High throughput screening of PU-PEG formulation tolerability was conducted on L929 cells treated with media extracted from vial-cast 23% (w/v) DABCO-catalyzed PU-PEG gels synthesized in ACN and DMF; 46% (w/v), 23% (w/v), and 15% (w/v) DABCO-catalyzed PU-PEG gels synthesized in DMSO; and 23% (w/v) DBTDL-catalyzed PU-PEG gels synthesized in DMF; FOTS-coated, sheet-cast, 23% (w/v), and 18% (w/v) DABCO-catalyzed PU-PEG gels synthesized in DMSO for 24 hrs (Figure S11A) and 72 hrs (Figure S11B). Similar metabolic activity levels were observed for both time points. There was not a significant impact on cell metabolic activity from treatment with media extracted from any of the DABCO-catalyzed hydrogels, regardless of concentration, casting condition or solvent in the initial hydrogel casting, but there was a significant reduction in metabolic activity seen in cells treated with media extracted from DBTDL-catalyzed hydrogels. Each data point is the average of 2-10 replicates per run. Any runs from hydrogels from the same synthesis were averaged together. The standard deviations are marked for formulations where data was collected from gels from multiple syntheses.

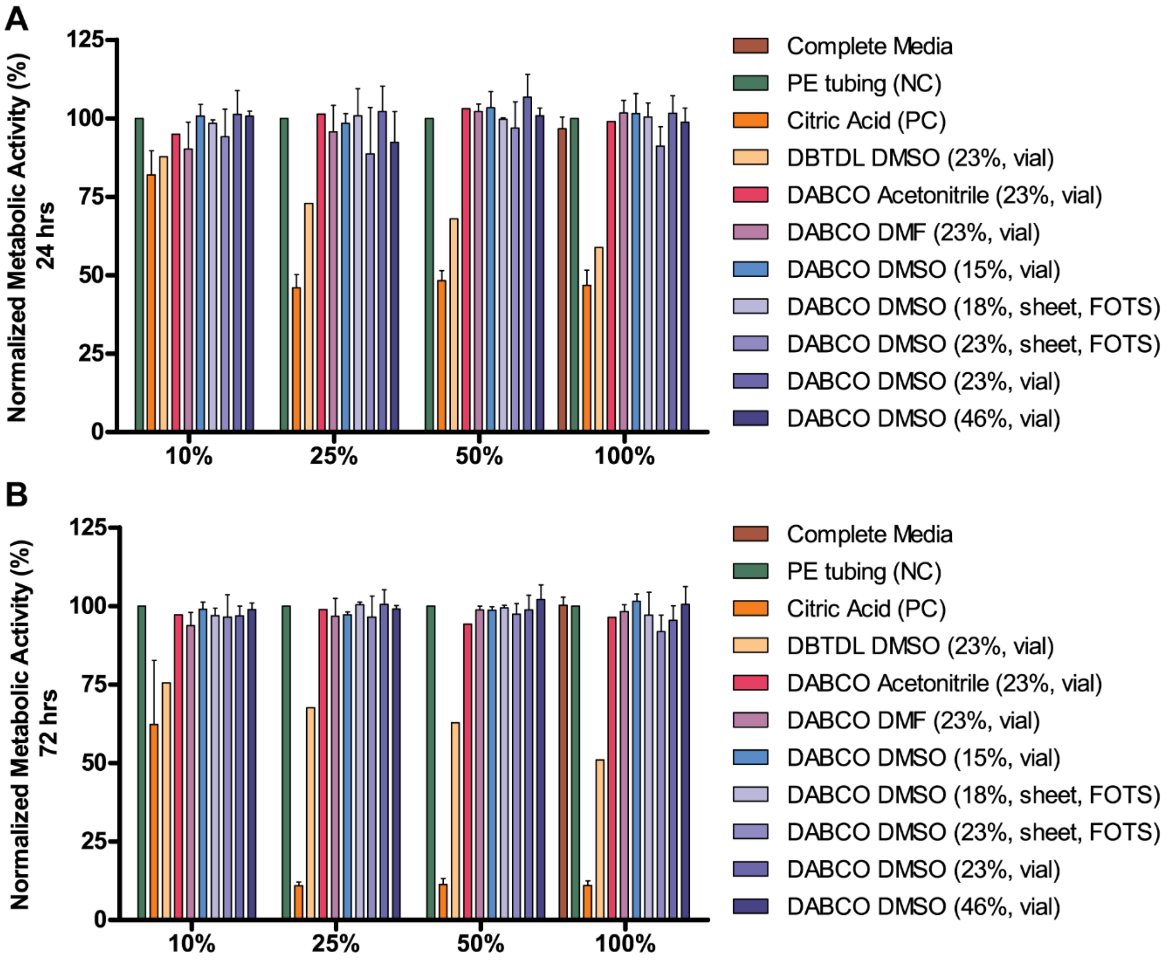

Figure S11. High throughput cytotoxicity screening of PU-PEG formulations: Normalized metabolic activity of L929 cells exposed to media treated with varying concentrations of complete media (red), polyethylene tubing (green), citric acid (orange), 23% (w/v) vial-cast DBTDL-catalyzed (yellow), 23% (w/v) vial-cast DABCO-catalyzed PU-PEG cast in ACN (pink), DMF (purple), 15% (w/v) vial-cast (blue), 18% (w/v) FOTS-coated sheet-cast (light purple), 23% (w/v) vial-cast (middle purple), FOTS-coated sheet-cast (purple), and 46% (w/v) vial-cast (dark purple) PU-PEG cast in DMSO after (A) 24 hrs or (B) 72 hrs assessed through Hoechst dye cell counting. Each data point represents the average of 2-10 replicates run from a single synthesis. If samples from more than two syntheses were included, an error bar representing the standard deviation is displayed.

To determine the most appropriate metabolic activity assay for the high throughput screening, cell quantification through a Hoechst nuclear dye (Figure S12A) and cell metabolic activity quantification through a CellTiter Glo assay (Figure S12B) were compared. L929 fibroblast cells exhibited little impact on their metabolic activity when treated with extracted media from vial-cast hydrogel formulations 23% (w/v) DABCO-catalyzed PU-PEG gels synthesized in acetonitrile and DMF; 46% (w/v), 23% (w/v), and 15% (w/v) DABCO-catalyzed PU-PEG gels synthesized in DMSO; and 23% (w/v) DBTDL-catalyzed PU-PEG gels synthesized in

DMF. The results were reasonably consistent for both methods, with some disagreement in the degree of the impact of DBTDL DMSO gels on the metabolic activity of treated cells at the 10% concentration. For the larger screening, the Hoechst dye method was selected due to lower variation in the data. Data collected from a single run of the screen, averages and standard deviations are representative of at least 3 replicates.

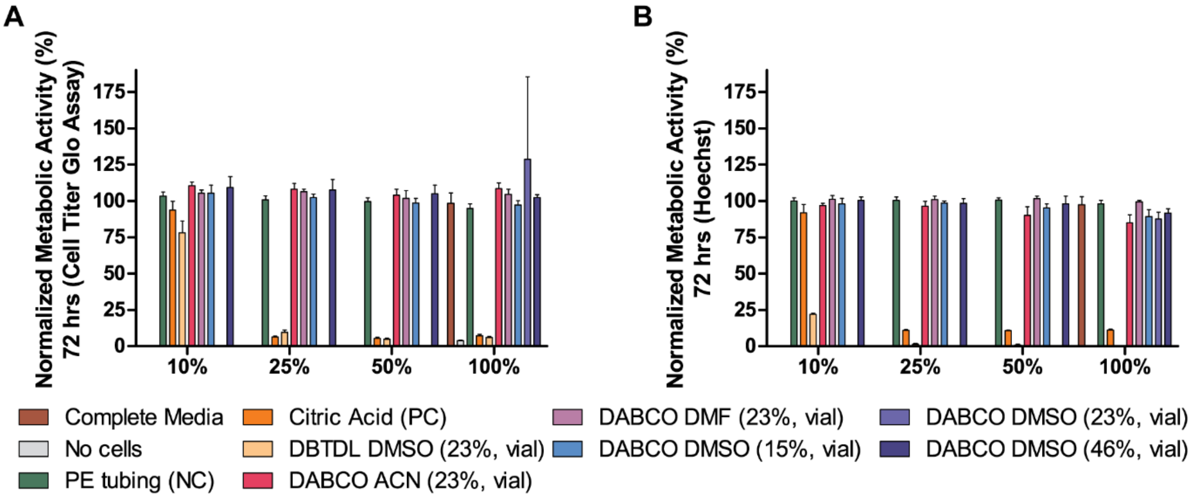

Figure S12. High throughput assay selection for cytotoxicity screening: Normalized metabolic activity of L929 cells exposed to media treated with varying concentrations of complete media (red), no cells (grey), polyethylene tubing (green), citric acid (orange), along with vial-cast 23% (w/v) DBTDL-catalyzed PU-PEG cast in DMSO, 23% (w/v) DABCO-catalyzed PU-PEG cast in ACN (pink), DMF (purple), 15% (w/v) (blue), 23% (w/v) (light purple), 46% (w/v) (dark purple) DABCO-catalyzed PU-PEG cast in DMSO and after 72 hrs assessed through (A) CellTiter-Glo metabolic activity assay and (B) Hoechst dye cell counting. Results represent the average of at least 3 replicates from a single synthesis.

The pH of the extracted media used in the cytotoxicity experiments was examined with a pH meter at room temperature, normal atmospheric CO_2 concentration. In the examination of casting conditions and synthesis catalyst, the extracted media from all PU-PEG hydrogel samples showed similar pH values compared with the complete media and polyethylene tubing controls (all were between pH 8-8.5, Figure S13A). Thus, the toxicity of the vial-cast DBTDL-catalyzed gels is not due to any deviation of pH value.

Similarly, the extraction of the PU-PEG samples incubated in water or blue food coloring (BFC) into media did not have a significant deviation of pH. The complete media and

polyethylene tubing controls exhibited a pH between 8.0-9.0, while the PU-PEG samples in water had a pH between 8.1-9.0 and the PU-PEG in BFC were between 7.5-7.9 (Figure S13B).

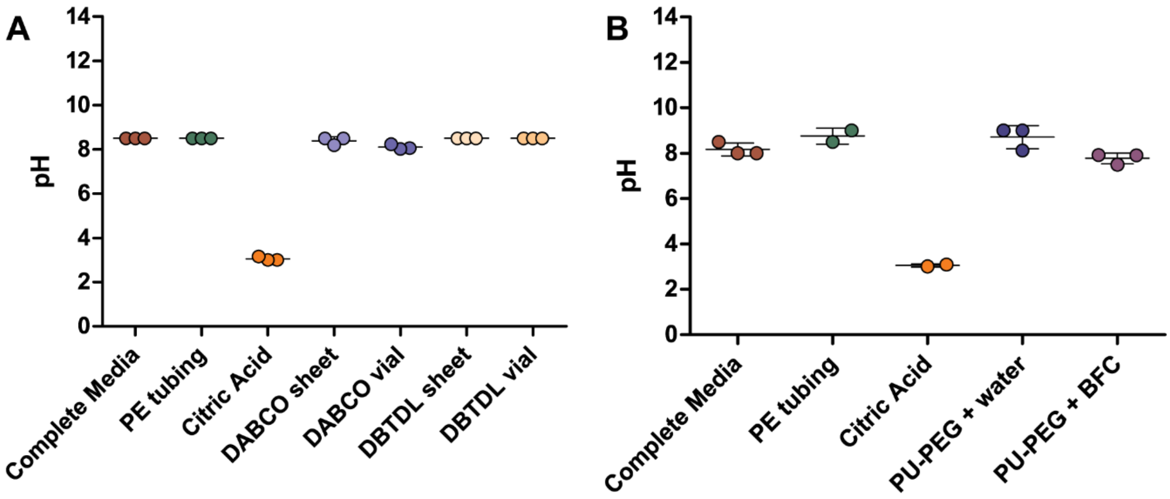

Figure S13. PU-PEG sample preparation for cytotoxicity testing. pH meter readings of extracted media samples for internal negative control, complete media (red), negative control polyethylene tubing (green), positive control citric acid (orange), (A) 23% (w/v) PU-PEG DABCO-catalyzed cast in a sheet (light blue) and vial configuration (dark blue), and 23% (w/v) PU-PEG DBTDL-catalyzed cast in a sheet (light gold) and vial configuration (dark gold) in the catalyst and casting condition experiments and (B) PU-PEG in water (blue) and PU-PEG in blue food coloring (BFC) (purple) in testing the biocompatibility of blue food coloring infiltration.

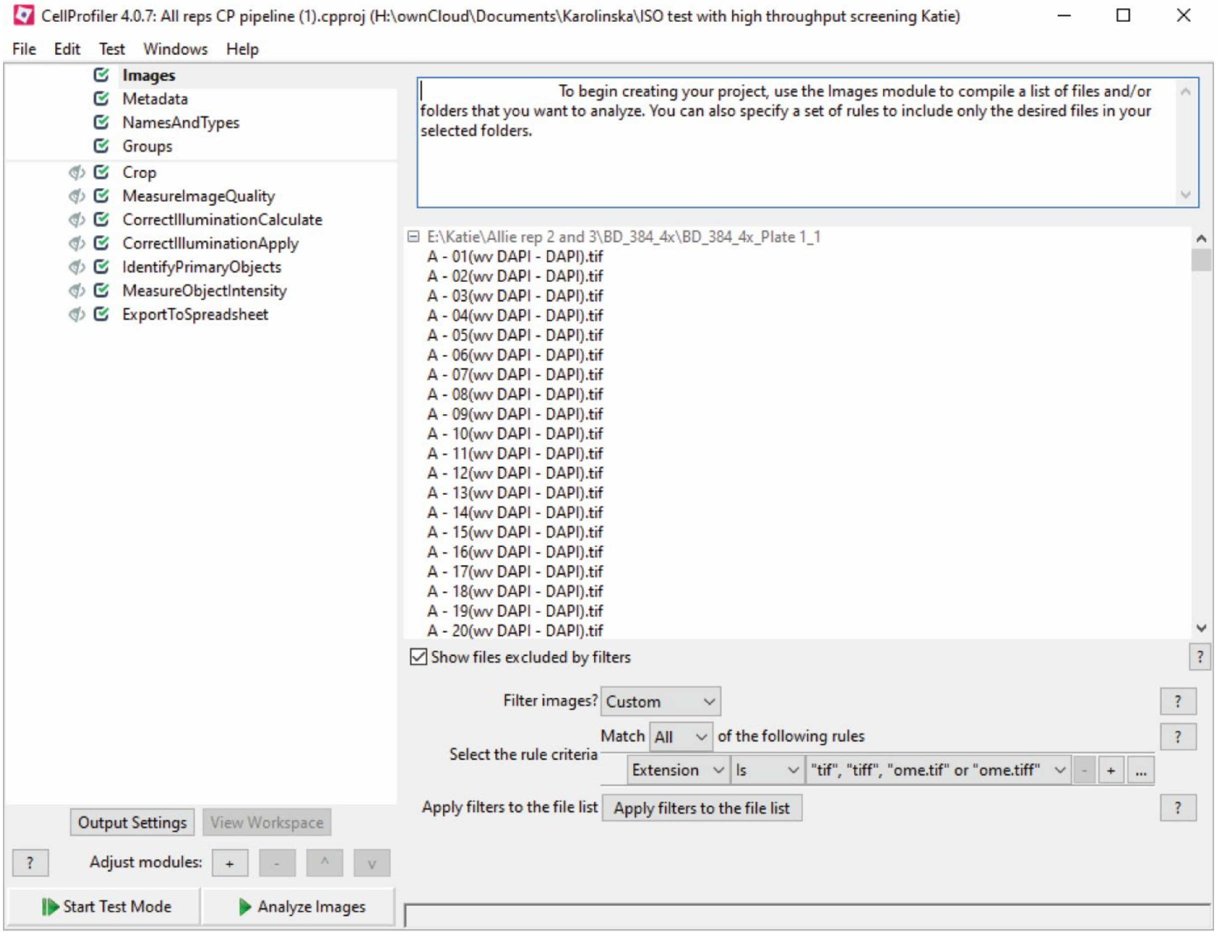

Figure S14. CellProfiler pipeline: Screenshot of representative workflow image analysis of cytotoxicity screening conducted in CellProfiler.

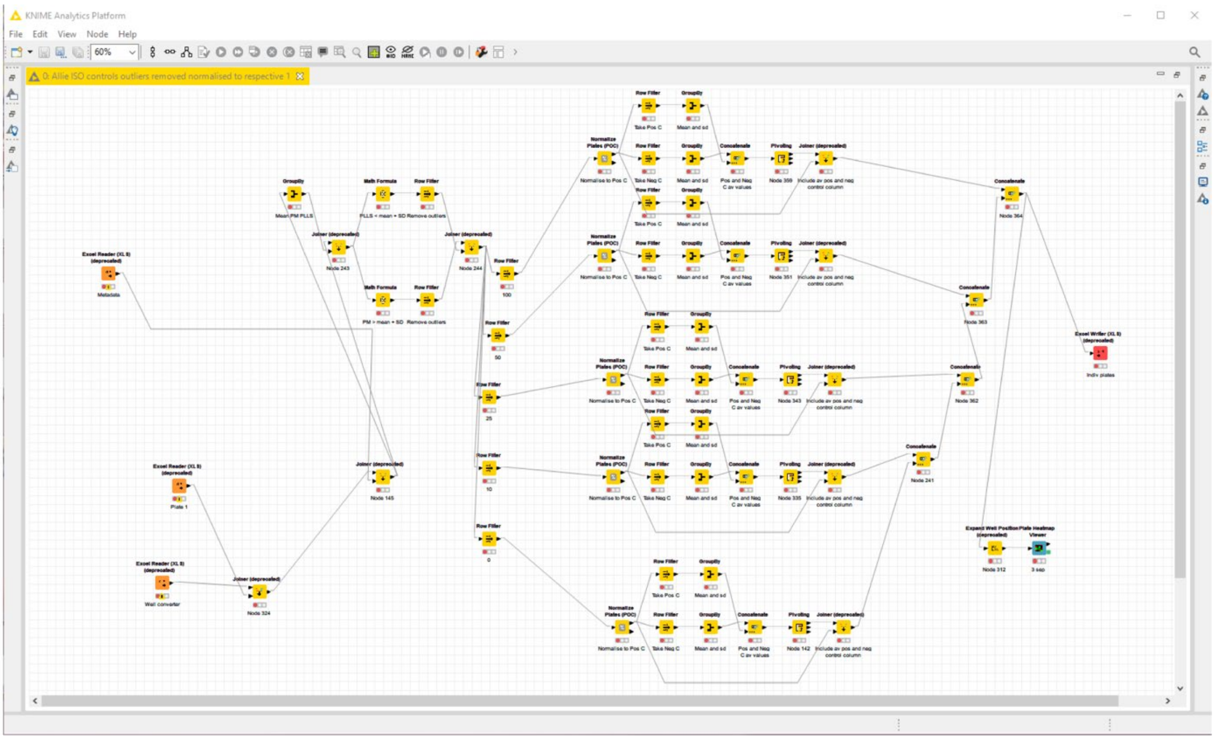

Figure S15. KNIME pipeline: Screenshot of representative workflow image analysis of cytotoxicity screening conducted in KNIME.

Calibration curves from bicinchoninic acid (BCA) assay correlating the absorbance at 562 nm of 23% (w/v) DABCO-catalyzed PU-PEG hydrogels with the concentration of albumin (Figure S16A) and fibrinogen (Figure S16B) solutions the gels are immersed in (0-1500 µg/mL). Calibration curves were determined from data points collected from 3 independent experiments, each run on at least three PU-PEG hydrogels from an independent synthesis. Curves were fit to the data points and used to extrapolate the concentration of protein absorbed to the experimental samples from their absorbance signal and are presented in Figure 4A.

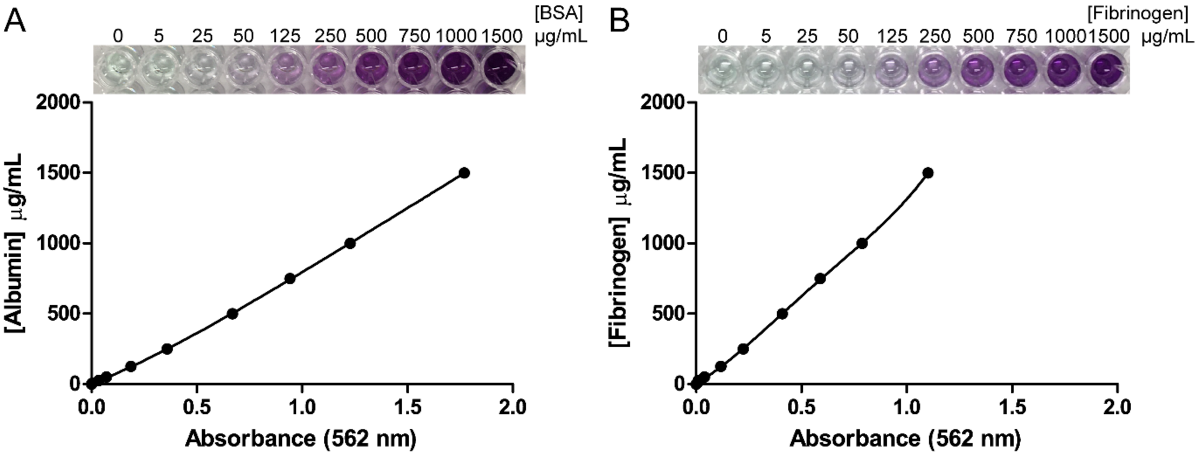

Figure S16. BCA assay calibration curves for further quantification of PU-PEG implant tolerability. Calibration curves for BCA assays correlating absorbance signal (562 nm) to (A) albumin and (B) fibrinogen concentrations (µg/mL) in 23% (w/v) DABCO-catalyzed PU-PEG hydrogels. Representative images of wells from calibration curve conducted in 96-well plate displayed above plots. Data points represent mean and standard deviation of 3 repeated experiments each containing 3 replicates, n = 3-4.

The foreign body response and long-term tolerability of the PU-PEG materials was assessed through implantation around the vagus nerve in a mouse model. Hydrogels were sterilized through two washes in 70% (v/v) ethanol and then rehydrated with six additional washes in ultrapure water. Gels were immersed overnight in filter-sterilized blue food coloring to facilitate identification of the gel from the surrounding tissue at the harvest time points. The PU-PEG gels were cut along their radius with a scalpel and punctured with a 27G needle (to encompass the diameter of the vagus nerve) to facilitate placement around the vagus nerve

(Figure S17A). The endotoxin content of the prepared hydrogels was assessed with a Chromogenic LAL assay (Figure S17E) and was consistently determined to be well below the threshold recommended by the FDA and significantly below the generally reported threshold for research-grade reagents (1 EU/mL).

The vagus nerve was exposed through separation of the salivary glands of the mouse (Figure S17B). The PU-PEG gels were sutured in place around the vagus nerve (Figure S17C). After 14 days and 41 days, the mice were perfused with PBS and PFA. The heads of the mice were collected and placed in PFA. Heads were then placed in a sucrose solution until fully infiltrated (indicated by sinking of the tissue into the sucrose solution). Heads were then opened up gently and the material along with the surrounding tissue was collected for embedding in OCT (Figure S17D).

The PU-PEG hydrogels stained with filter-sterilized blue food coloring were assessed according to the ISO standards. Cells treated with media extracted from PU-PEG hydrogels hydrated in ultrapure water and PU-PEG saturated with blue food coloring showed no statistically significant impact on cell metabolic activity at 24 hrs (Figure S17F) or 72 hrs (Figure S17G).

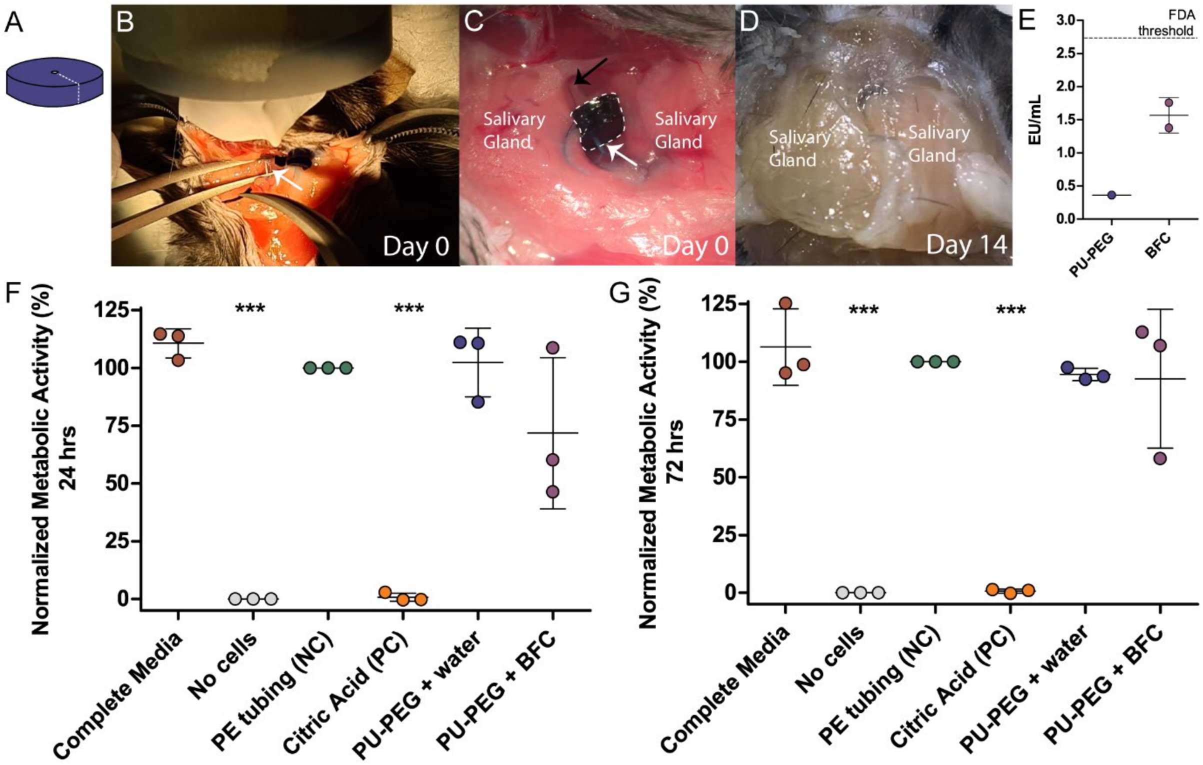

Figure S17. Implantation method for assessing long-term PU-PEG tolerability. (A) PU-PEG hydrogels were stained with blue food coloring to facilitate their identification at the long-term time points and differentiate from the surrounding tissue. A scalpel was used to cut along the radius of the gel and a 27G needle removed ~200 µm from the center of the gel to fit around the vagus nerve. (B) Salivary glands were separated to expose the right vagus nerve and (C) PU-PEG hydrogel was placed around the nerve and sutured in place. White arrows point to the vagus nerve and the black arrow points to the suture used to hold the gel in place. White dotted line highlights the PU-PEG gel. (D) The appearance of the material and surrounding tissue after harvest, fixation, and sucrose immersion, just before sample embedding. (E) Endotoxin quantification of representative PU-PEG hydrogel and blue food coloring used in the long-term implant study. Normalized metabolic activity of L929 cells exposed to media treated with varying concentrations of polyethylene tubing (green), no cells (gray), complete media (red), citric acid (orange), PU-PEG in water (blue) and PU-PEG in blue food coloring (BFC) (purple) after (F) 24 hrs or (G) 72 hrs. Metabolic activity normalized to polyethylene tubing (green) for each experimental repeat. Internal controls where L929 cells were grown in their traditional complete media (red) and where no cells were included in the L929 traditional complete media (grey) were included for experimental rigor. Each metabolic activity experiment conducted on gels from one of 3 independent synthesis ($n = 3$) is represented by a single data point, each experimental run composed of 3-5 replicates. Error bars are standard deviation. One-way ANOVA comparisons between conditions conducted with Tukey post-test, *** $p < 0.0001$ compared with PE tubing, complete media, PU-PEG in water and PU-PEG in blue food coloring (BFC) samples.

References

[1] A. Tabet, S. Mommer, J. A. Vigil, C. Hallou, H. Bulstrode, O. A. Scherman, *Adv. Healthc. Mater.* **2019**, *8*.
[2] M. Stolz, R. Raiteri, A. U. Daniels, M. R. VanLandingham, W. Baschong, U. Aebi, *Biophys. J.* **2004**, *86*, 3269.
[3] H. T. Nia, I. S. Bozchalooi, Y. Li, L. Han, H. H. Hung, E. Frank, K. Youcef-Toumi, C.

Ortiz, A. Grodzinsky, *Biophys. J.* **2013**, *104*, 1529.
